# Supplementary material for: Synthesis, Characterization, and Biological Activity of a Novel Series of Benzo[4,5]imidazo[2,1-b]thiazole Derivatives as Potential Epidermal Growth Factor Receptor Inhibitors
Source: Molecules. 2019 Feb 14;24(4):682. doi: 10.3390/molecules24040682 (PMC6412895; doi:10.3390/molecules24040682)
Supplement: Supplementary file 1 [file molecules-24-00682-s001.pdf]

molecules

Supplementary Materials

Synthesis, Characterization and Biological Activity of a Novel Series of Benzo[4,5]imidazo[2,1-b]thiazole Derivatives as Potential Epidermal Growth Factor Receptor Inhibitors

Xinshan Deng [1], Xiaoyu Tan [1], Tiantian An [1], Qingqing Ma [1], Zhe Jin [1,*], Ce Wang [1], Qingguo Meng [2], Chun Hu [2,*]

[1] Key Laboratory of Structure-Based Drug Design & Discovery, Ministry of Education; Shenyang Pharmaceutical University, Shenyang 110016, China; E-Mails: xinshandeng@126.com (X. Deng); tanxiaoyuaikaoyan@163.com (X. Tan); antiantiansyphu@163.com (T. An); maqingqing01@163.com (Q. Ma); jinzheln@163.com (Z. Jin); wangce0412@163.com (C. Wang); chunhu@syphu.edu.cn (C. Hu).

[2] Department of Pharmacy, Yantai Univeristy, Yantai 264005, China; E-Mail: qinggmeng@163.com (Q. Meng).

* Correspondences: jinzheln@163.com (Z. Jin) and chunhu@syphu.edu.cn (C. Hu); Tel.: +86-24-43520246.

molecules

MDPI

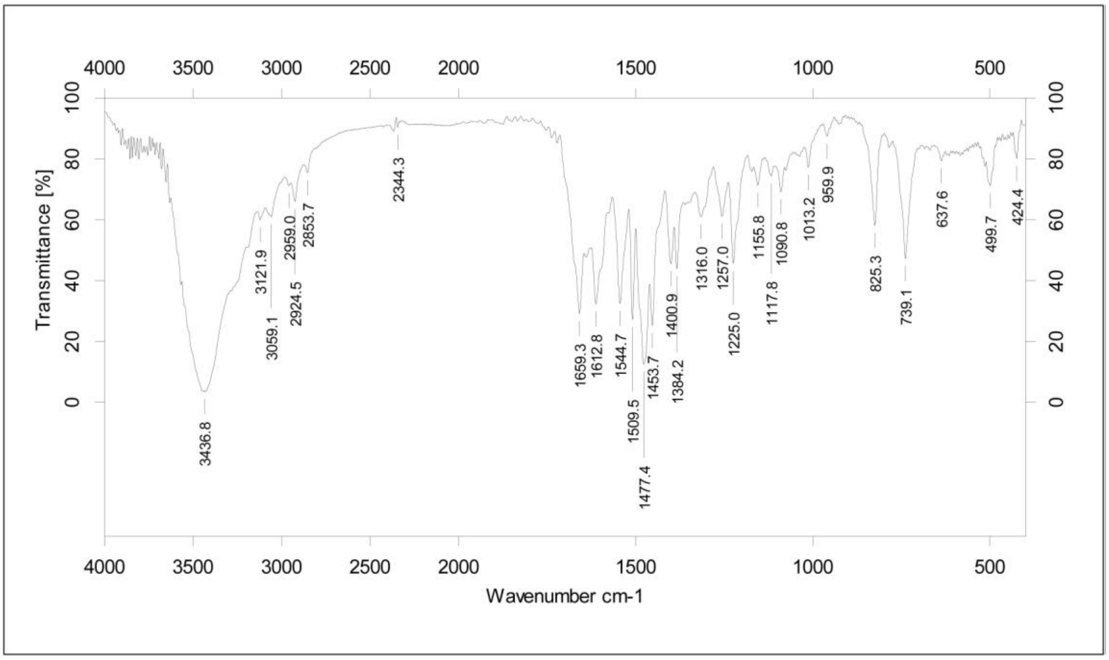

Sample : DXS-D-13	Frequency Range : 3999.64 - 400.157	Measured on : 2016-7-16	
Technique : Sample form	Resolution : 2	Instrument : EQUINOX55	Sample Scans : 16
Customer : Default	Zerofilling : 2	Acquisition : Double Sided,Forv	

Figure S01. IR spectra of the target compound D01

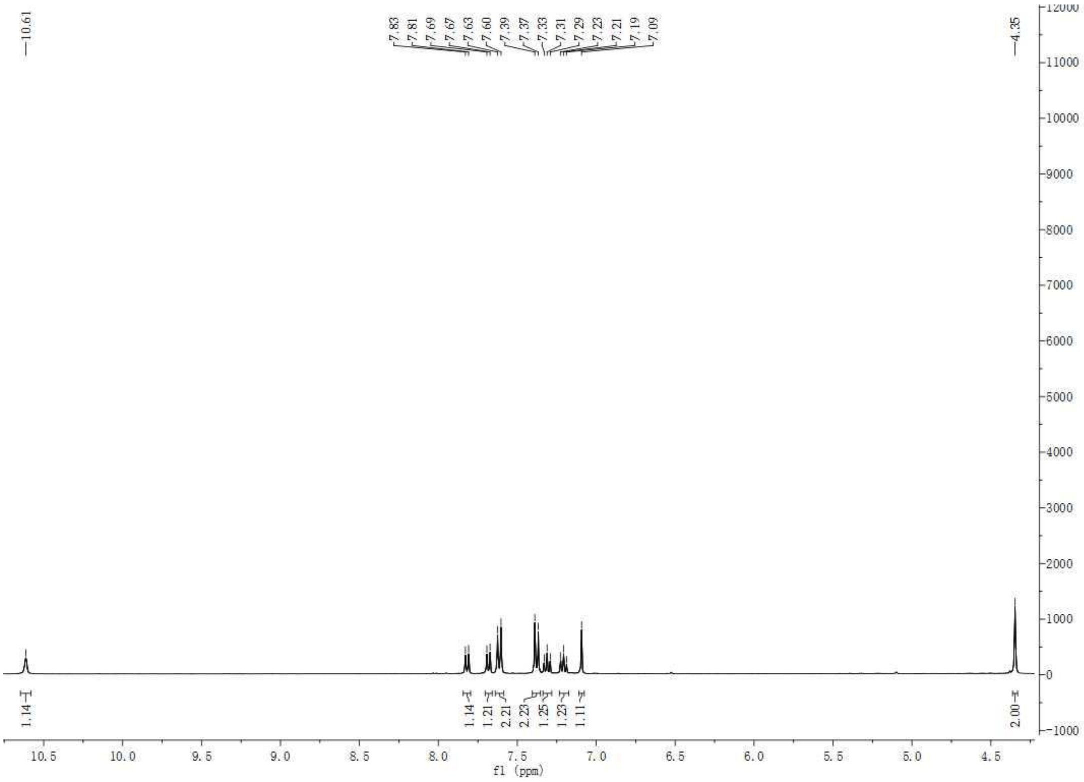

Figure S02. ^{1}H-NMR spectra of the target compound D01

molecules

MDPI

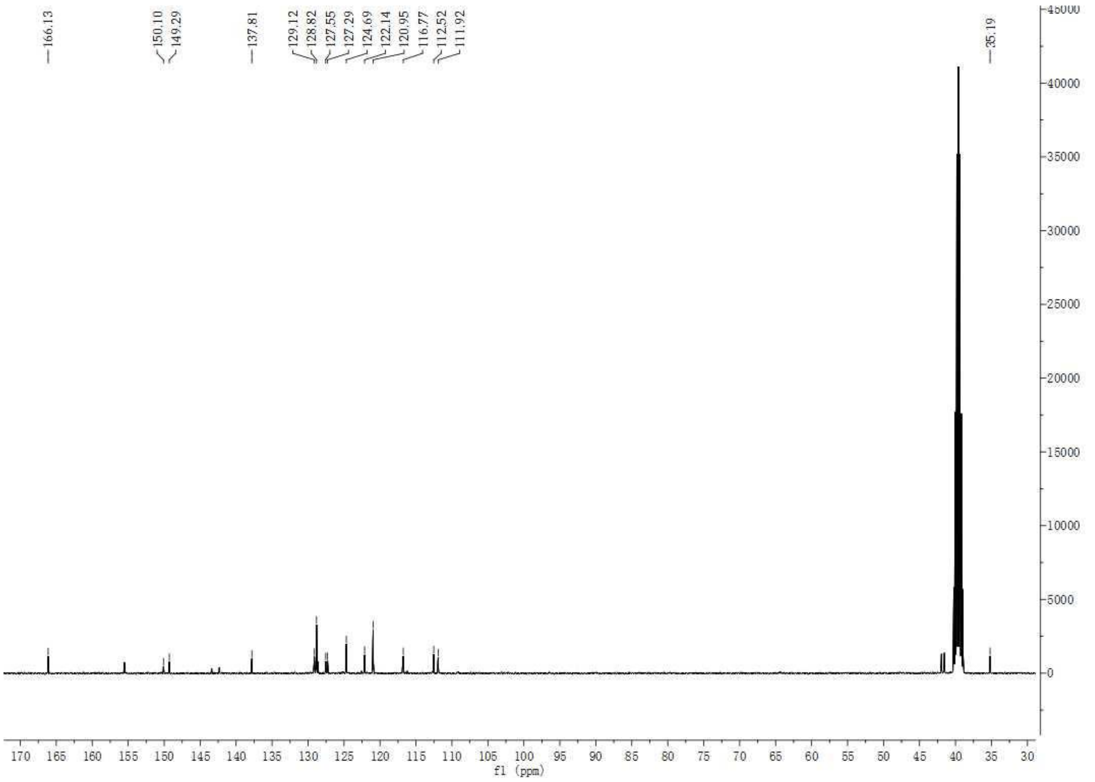

Figure S03. ^{13}C-NMR spectra of the target compound D01

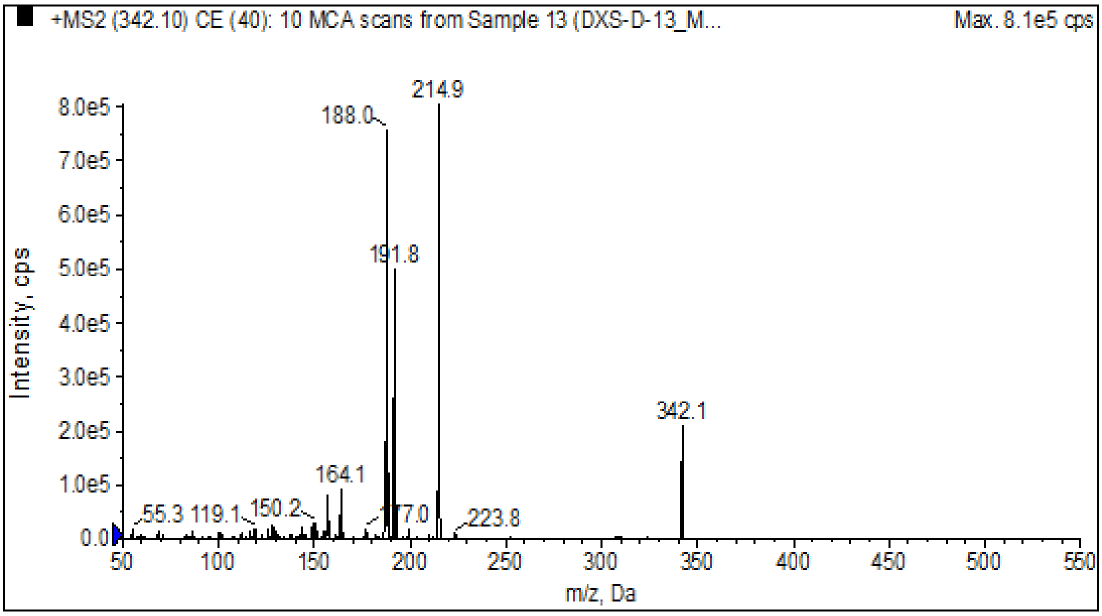

Figure S04. ESI-MS of the target compound D01

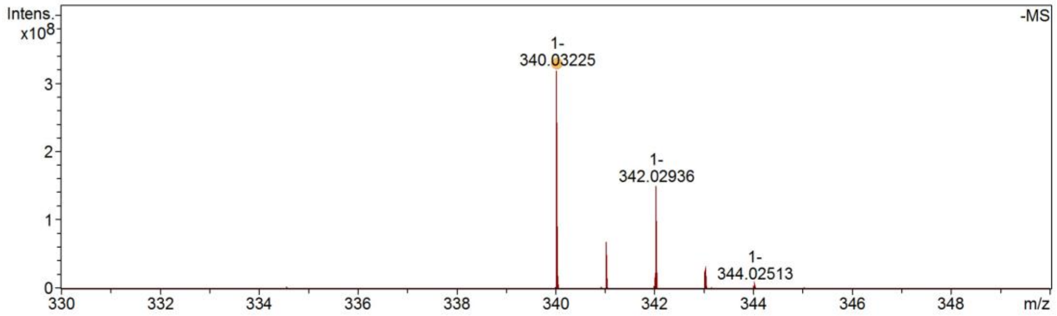

Meas. m/z	#	Ion Formula	Score	m/z	err [ppm]
340.032253	1	C17H11ClN3OS	100.00	340.031684	-1.7

Figure S05. HRMS of the target compound D01

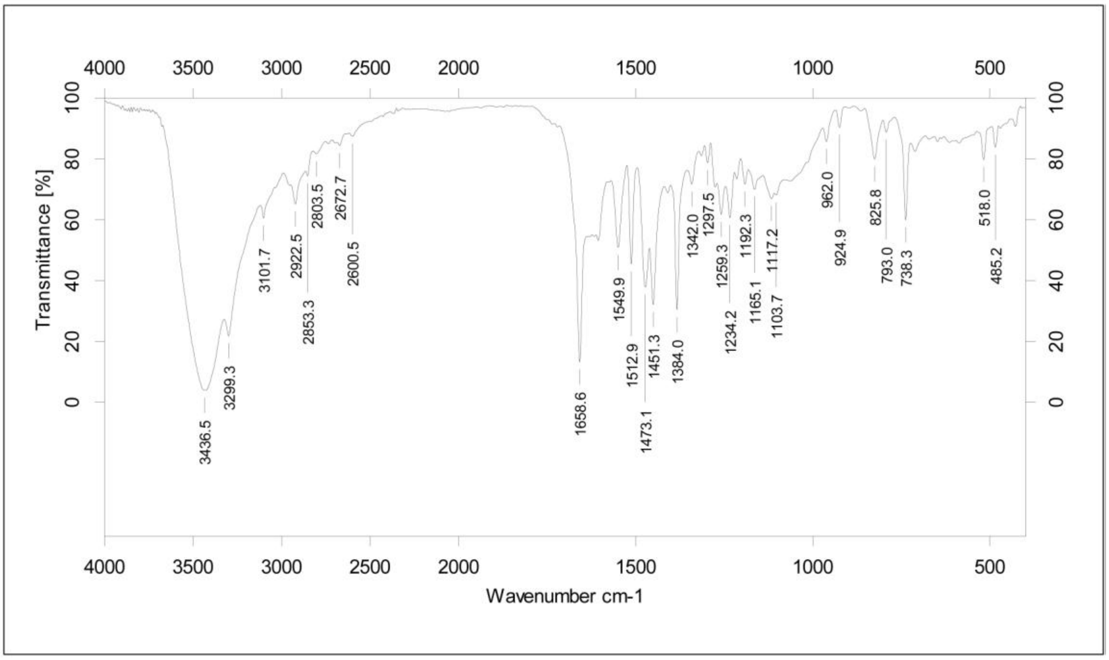

Sample : DXS-D-17	Frequency Range : 3999.64 - 400.157	Measured on : 2016-7-16	
Technique : Sample form	Resolution : 2	Instrument : EQUINOX55	Sample Scans : 16
Customer : Default	Zerofilling : 2	Acquisition : Double Sided,Forv	

Figure S06. IR spectra of the target compound D02

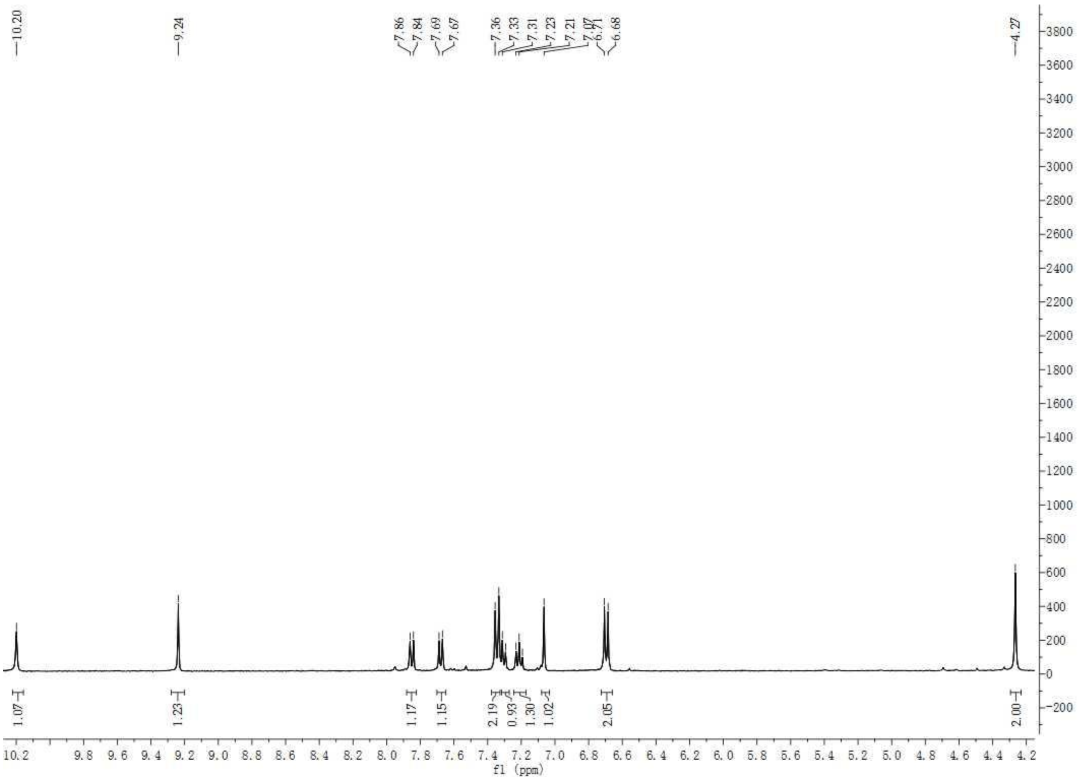

Figure S07. ^{1}H-NMR spectra of the target compound D02

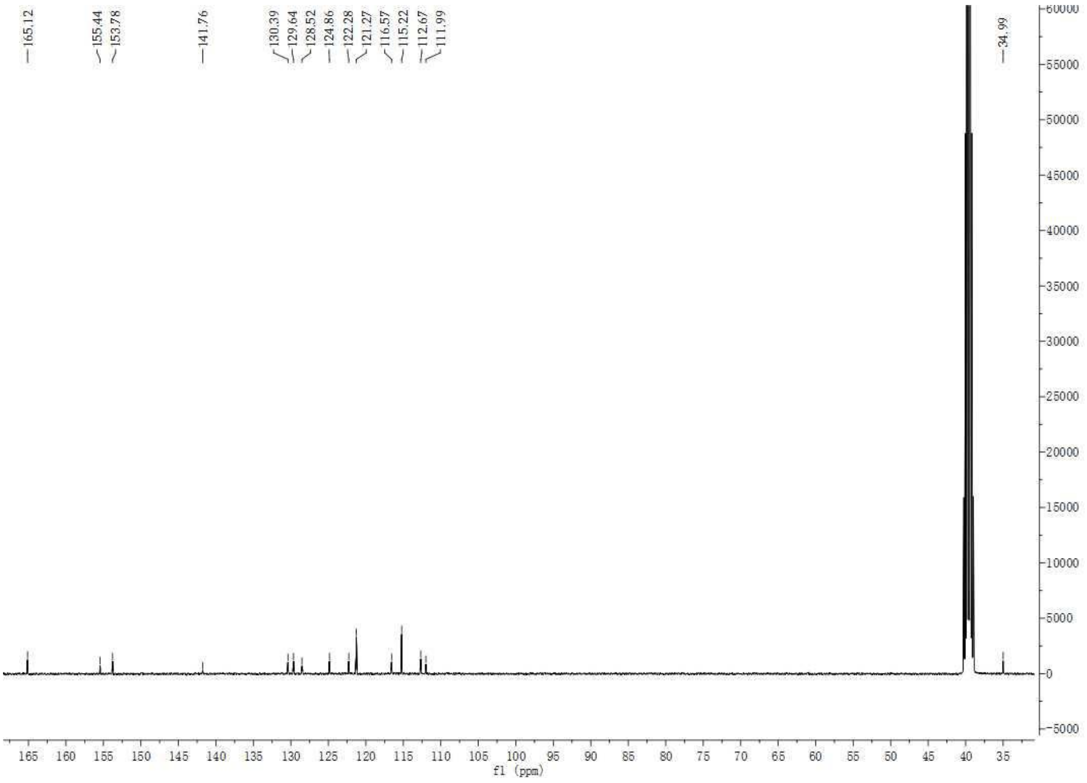

Figure S08. ^{13}C-NMR spectra of the target compound D02

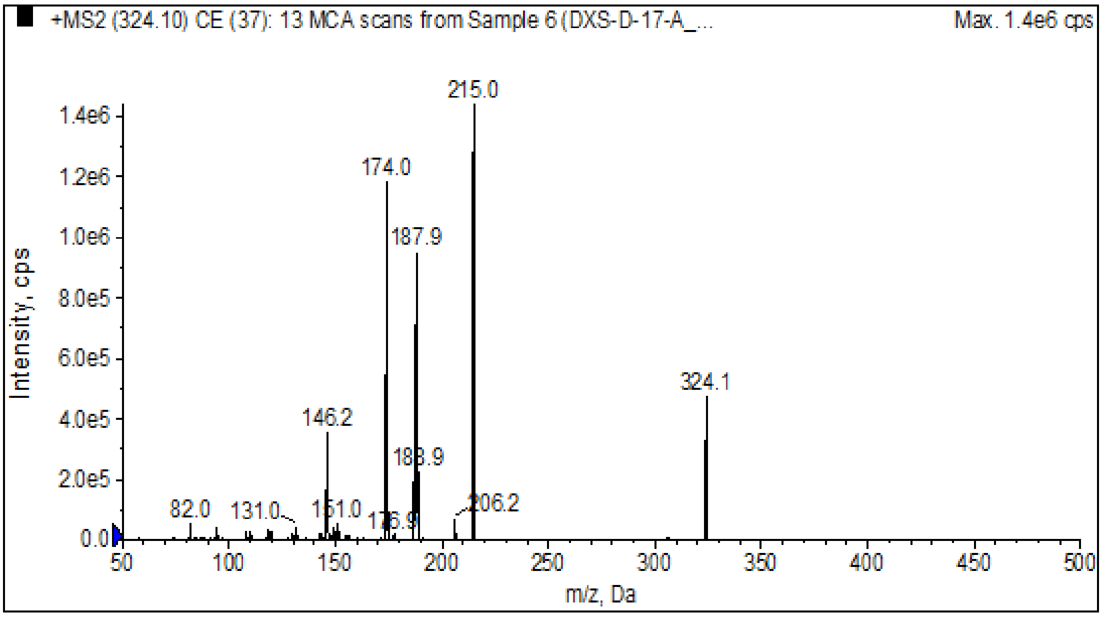

Figure S09. ESI-MS of the target compound D02

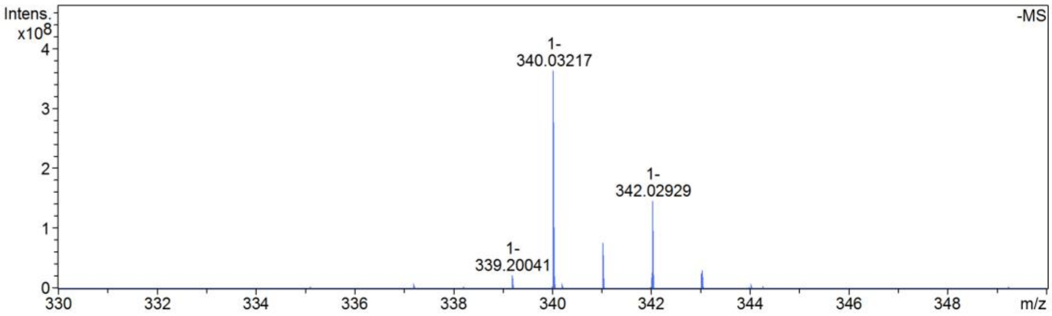

Meas. m/z	#	Ion Formula	Score	m/z	err [ppm]
322.066142	1	C17H12N3O2S	19.88	322.065571	-1.8

Figure S10. HRMS of the target compound D02

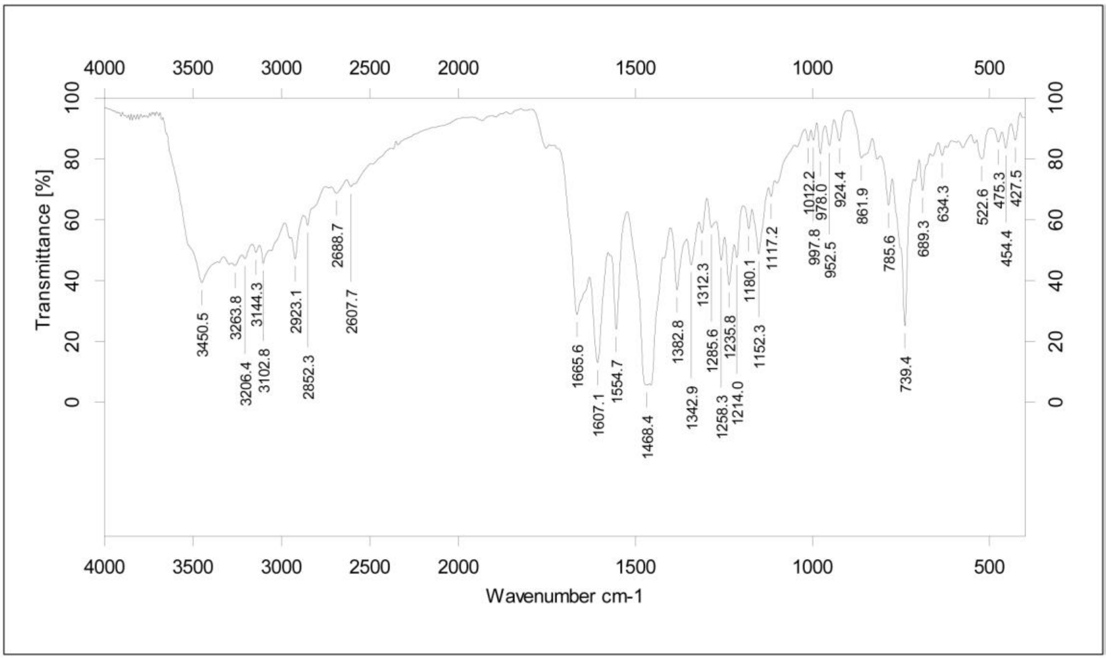

Sample : DXS-D-18	Frequency Range : 3999.64 - 400.157	Measured on : 2016-7-16	
Technique : Sample form	Resolution : 2	Instrument : EQUINOX55	Sample Scans : 16
Customer : Default	Zerofilling : 2	Acquisition : Double Sided,Forv	

Figure S11. IR spectra of the target compound D03

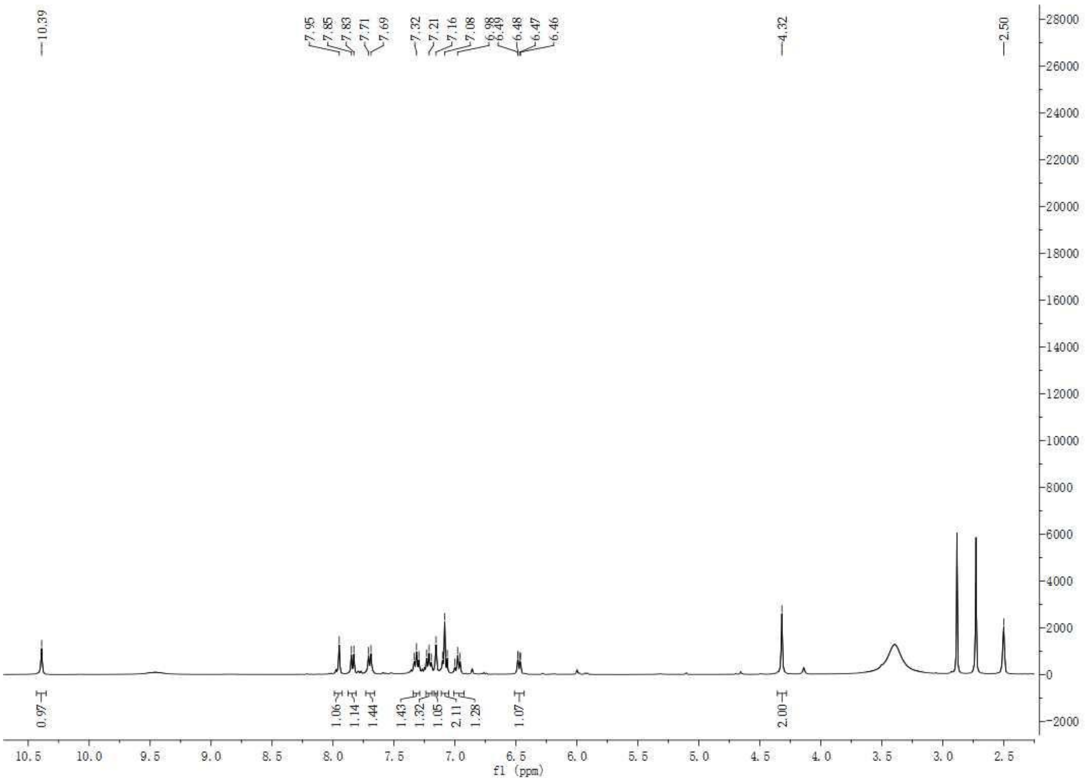

Figure S12. [1] H-NMR spectra of the target compound D03

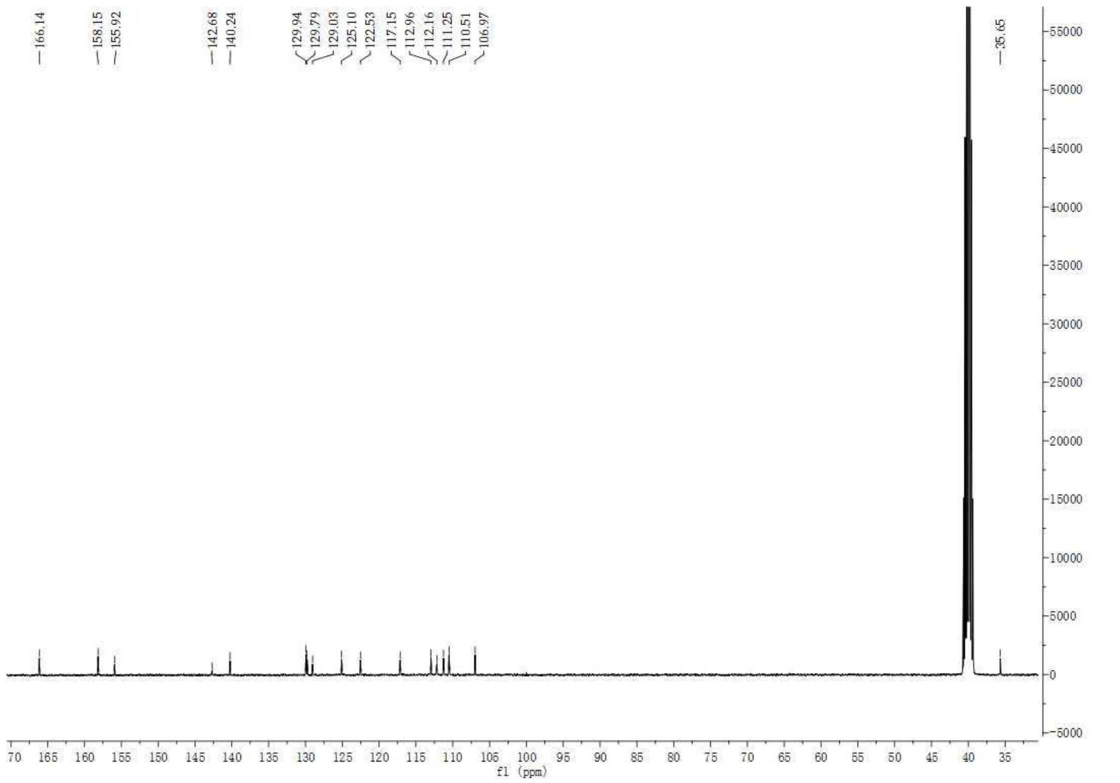

Figure S13. ^{13}C-NMR spectra of the target compound D03

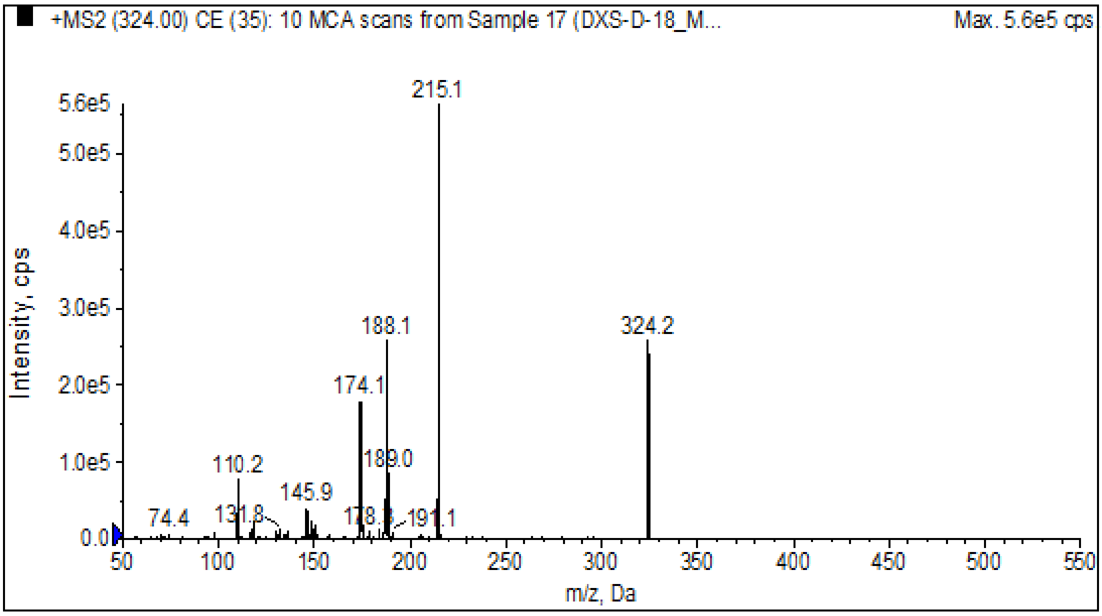

Figure S14. ESI-MS of the target compound D03

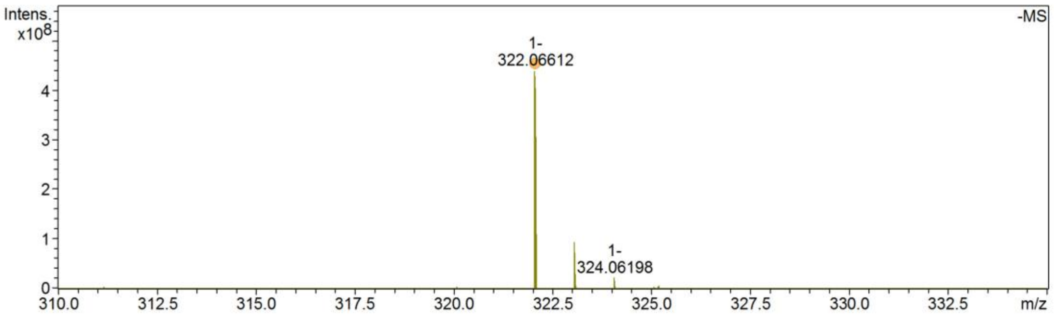

Meas. m/z	#	Ion Formula	Score	m/z	err [ppm]
322.066120	1	C17H12N3O2S	20.25	322.065571	-1.7

Figure S15. HRMS of the target compound D03

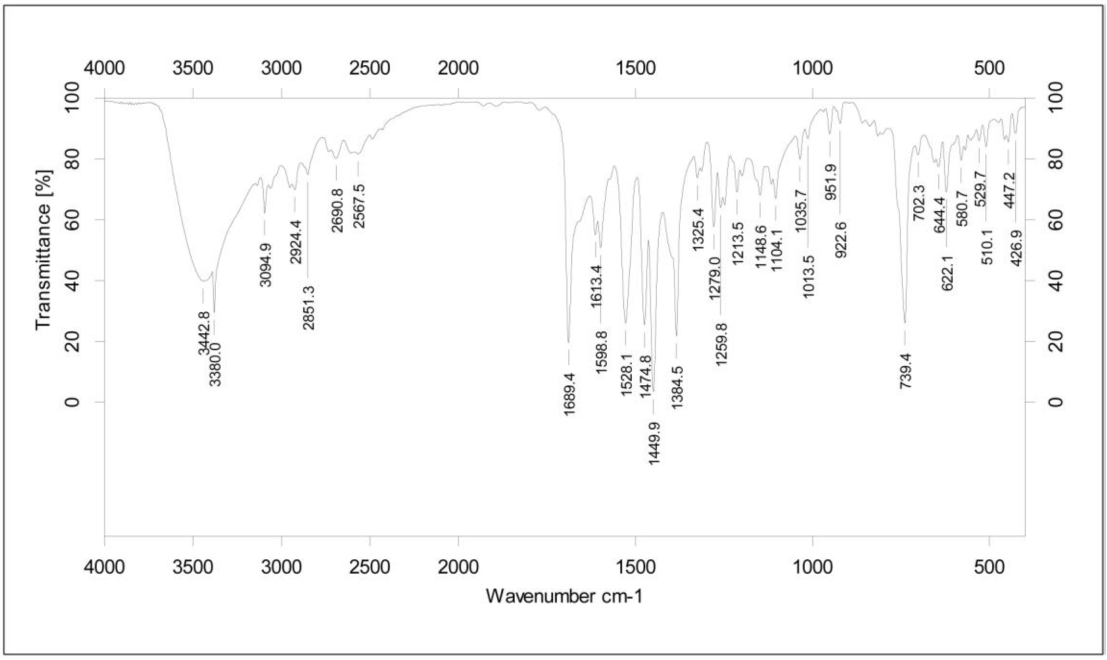

Sample : DXS-D-19	Frequency Range : 3999.64 - 400.157	Measured on : 2016-7-16	
Technique : Sample form	Resolution : 2	Instrument : EQUINOX55	Sample Scans : 16
Customer : Default	Zerofilling : 2	Acquisition : Double Sided,Forv	

Figure S16. IR spectra of the target compound D04

molecules

MDPI

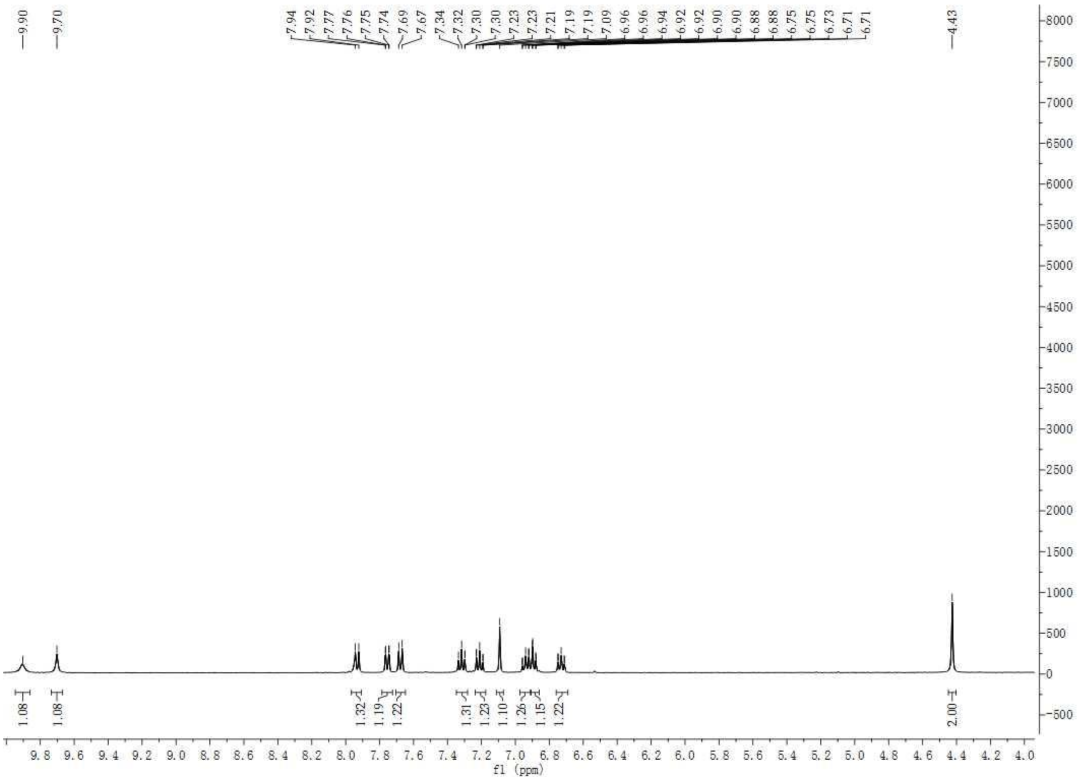

Figure S17. [1] H-NMR spectra of the target compound D04

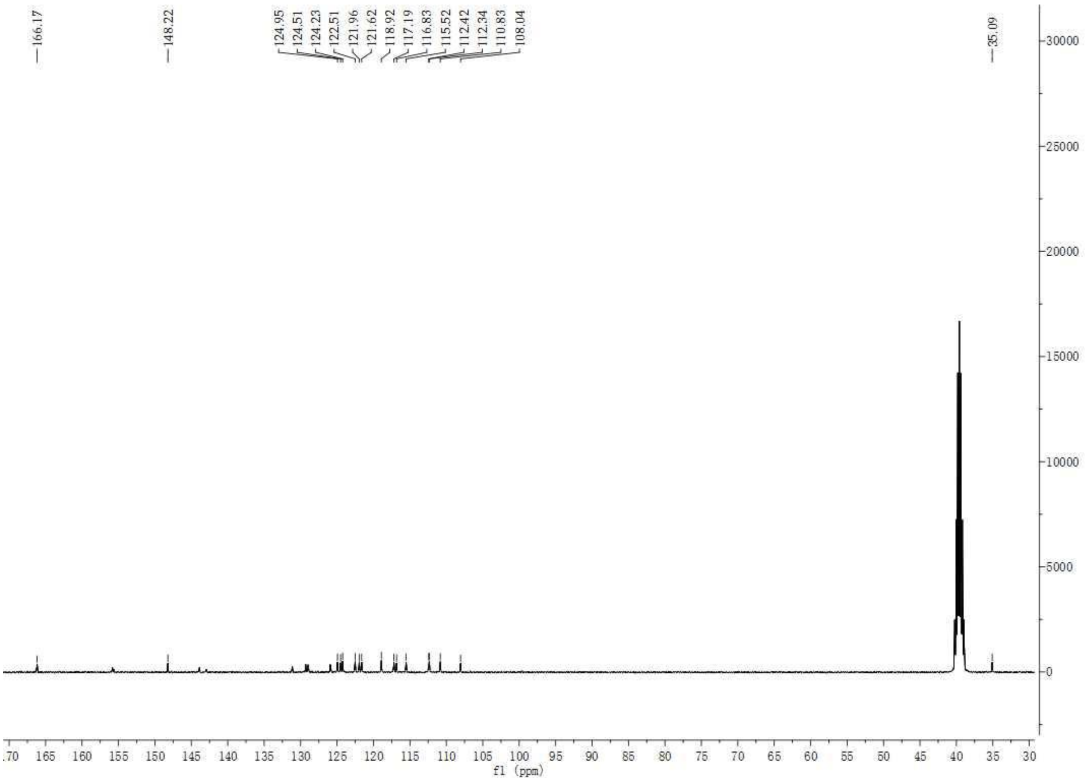

Figure S18. [13] C-NMR spectra of the target compound D04

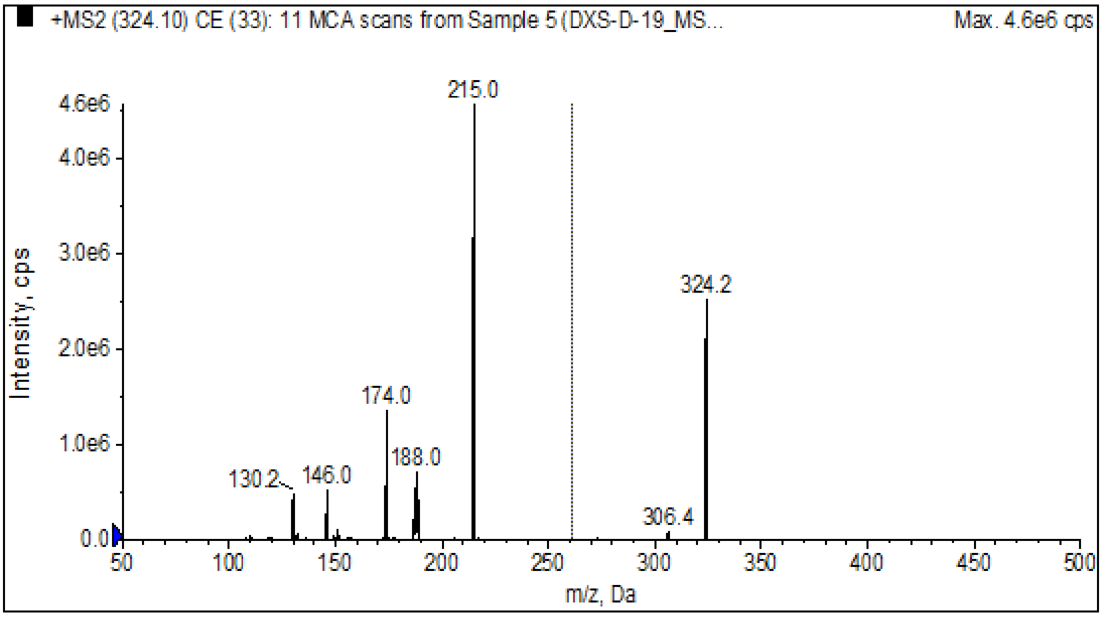

Figure S19. ESI-MS of the target compound D04

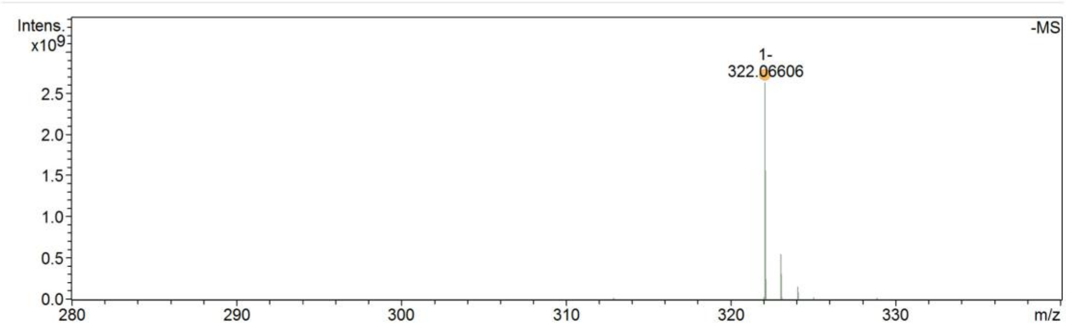

Meas. m/z	#	Ion Formula	Score	m/z	err [ppm]
322.066063	1	C17H12N3O2S	21.13	322.065571	-1.5

Figure S20. HRMS of the target compound D04

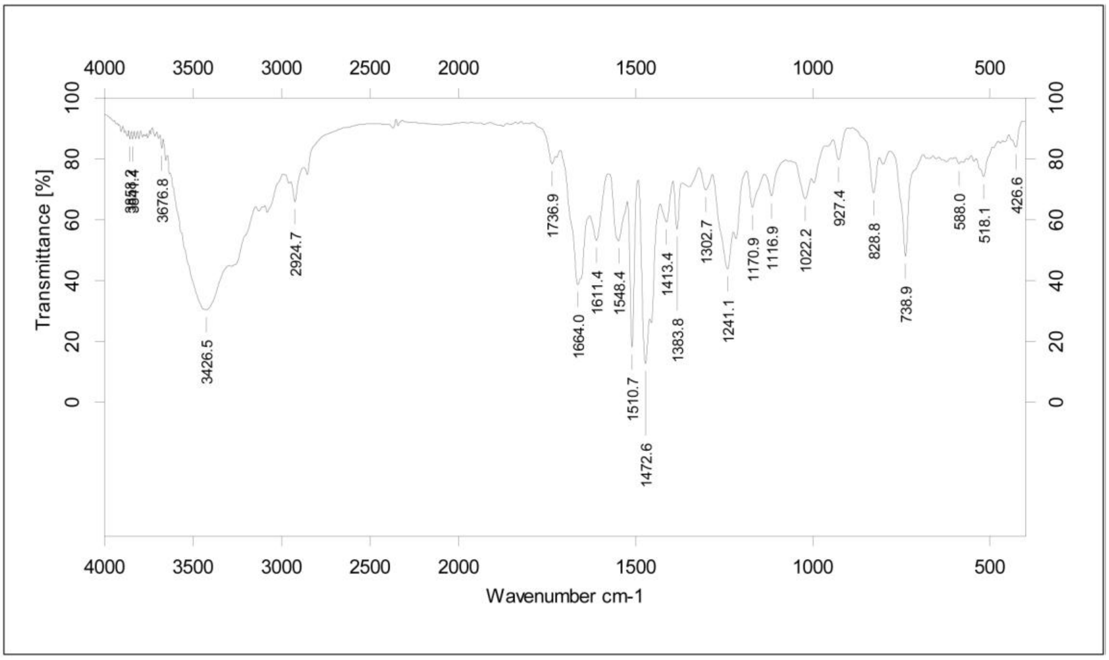

Sample : DXS-D-20	Frequency Range : 3999.64 - 400.157	Measured on : 2016-6-18	
Technique : Sample form	Resolution : 2	Instrument : EQUINOX55	Sample Scans : 16
Customer : Default	Zerofilling : 2	Acquisition : Double Sided,Forv	

Figure S21. IR spectra of the target compound D05

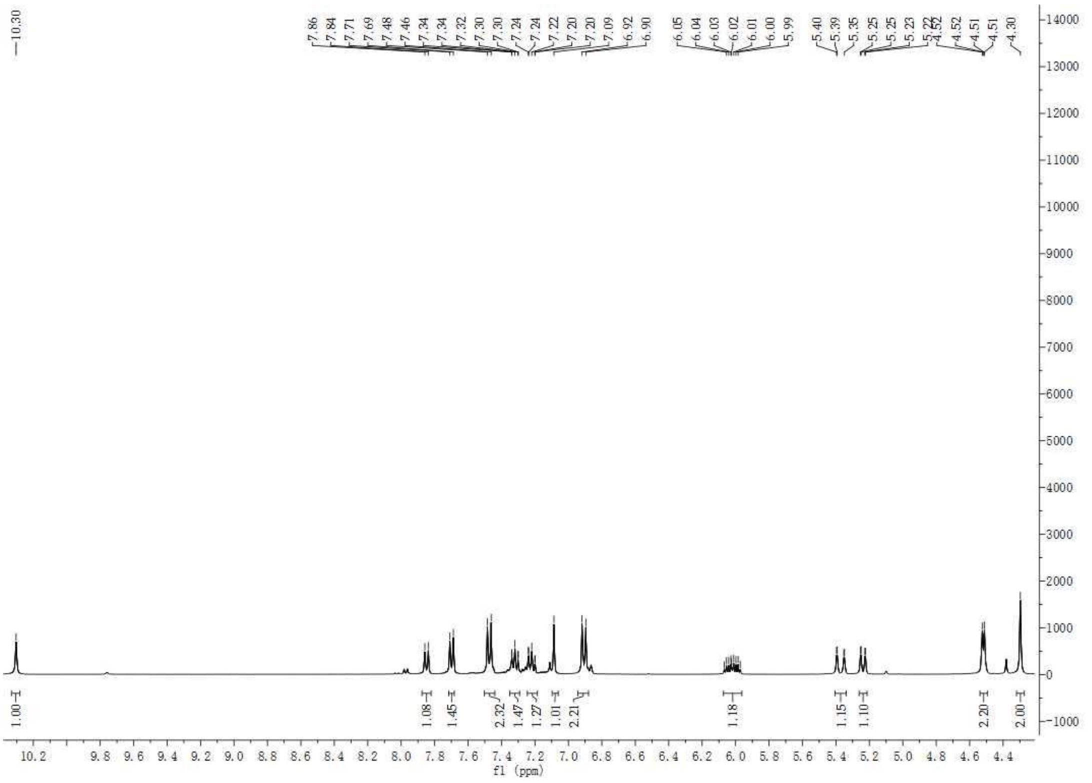

Figure S22. ^{1}H-NMR spectra of the target compound D05

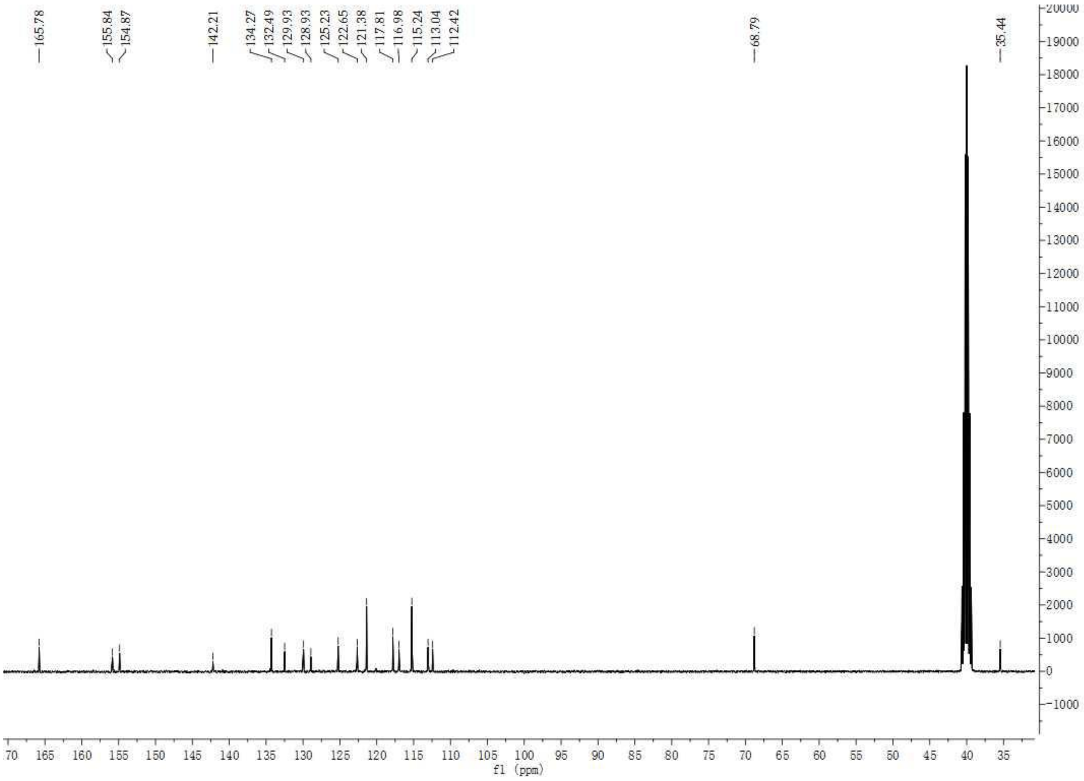

Figure S23. ^{13}C-NMR spectra of the target compound D05

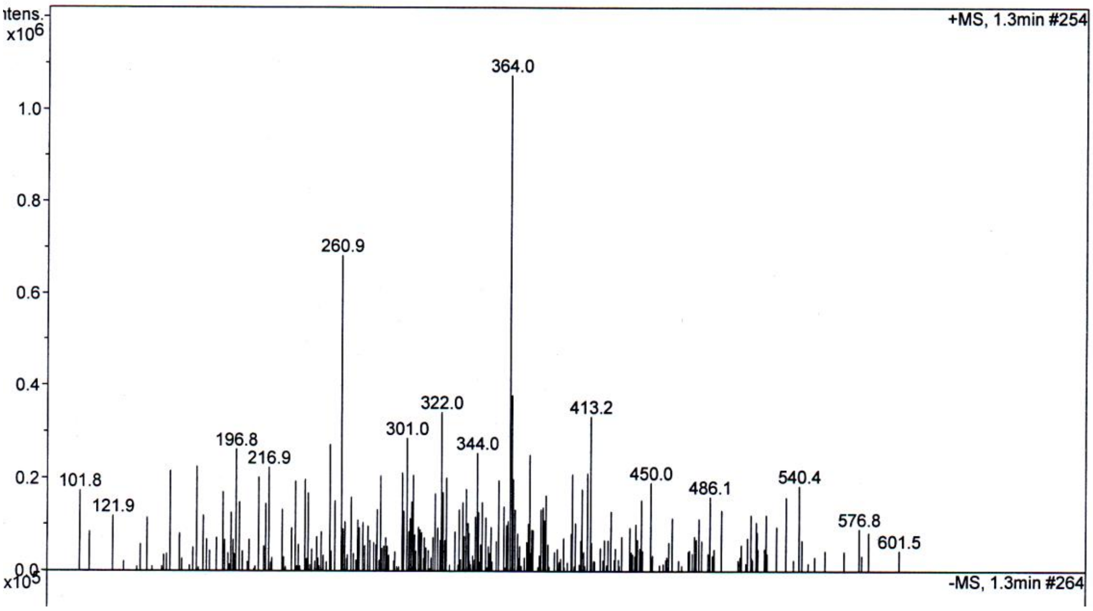

Figure S24. ESI-MS of the target compound D05

molecules MDPI

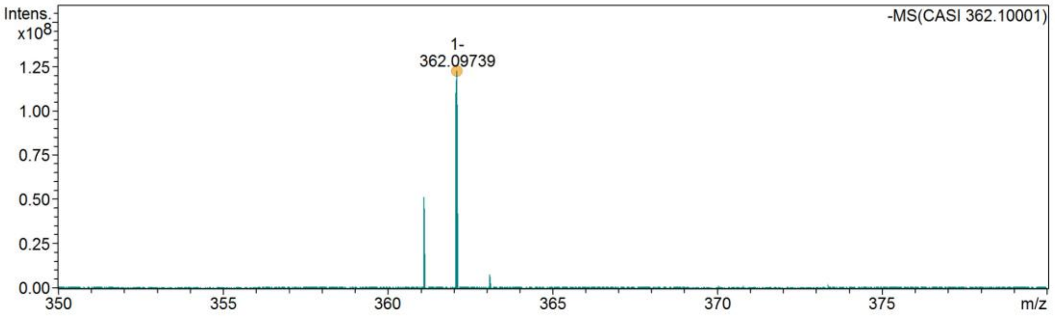

Meas. m/z	#	Ion Formula	Score	m/z	err [ppm]
362.097386	1	C20H16N3O2S	100.00	362.096871	-1.4

Figure S25. HRMS of the target compound D05

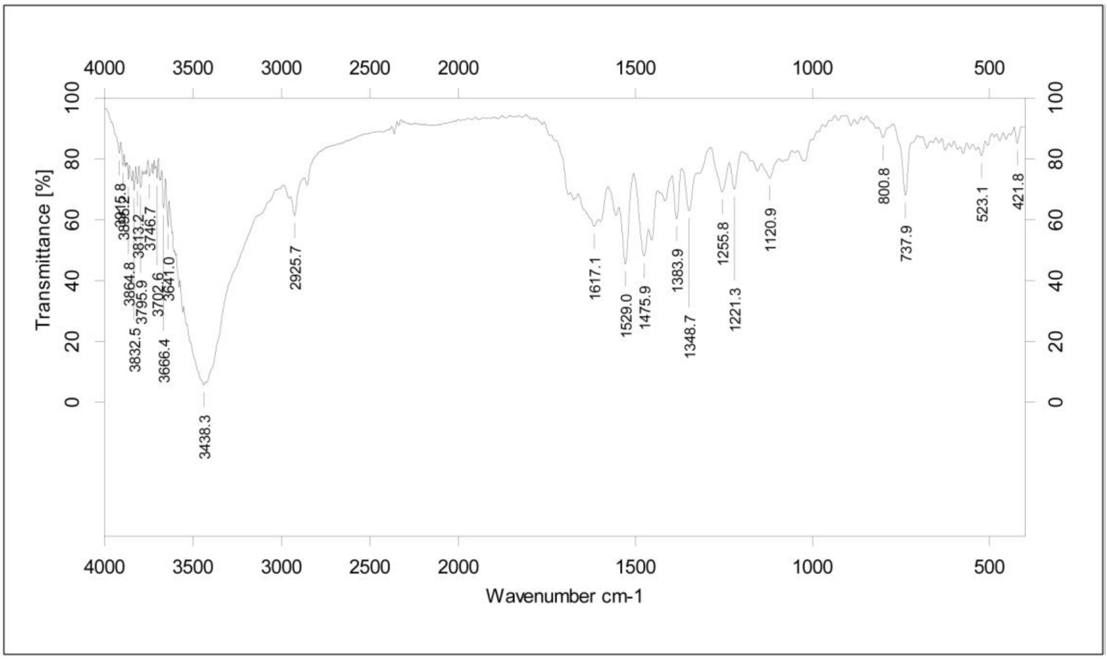

Sample : DXS-D-21	Frequency Range : 3999.64 - 400.157	Measured on : 2016-6-18	
Technique : Sample form	Resolution : 2	Instrument : EQUINOX55	Sample Scans : 16
Customer : Default	Zerofilling : 2	Acquisition : Double Sided,Forv	

Figure S26. IR spectra of the target compound D06

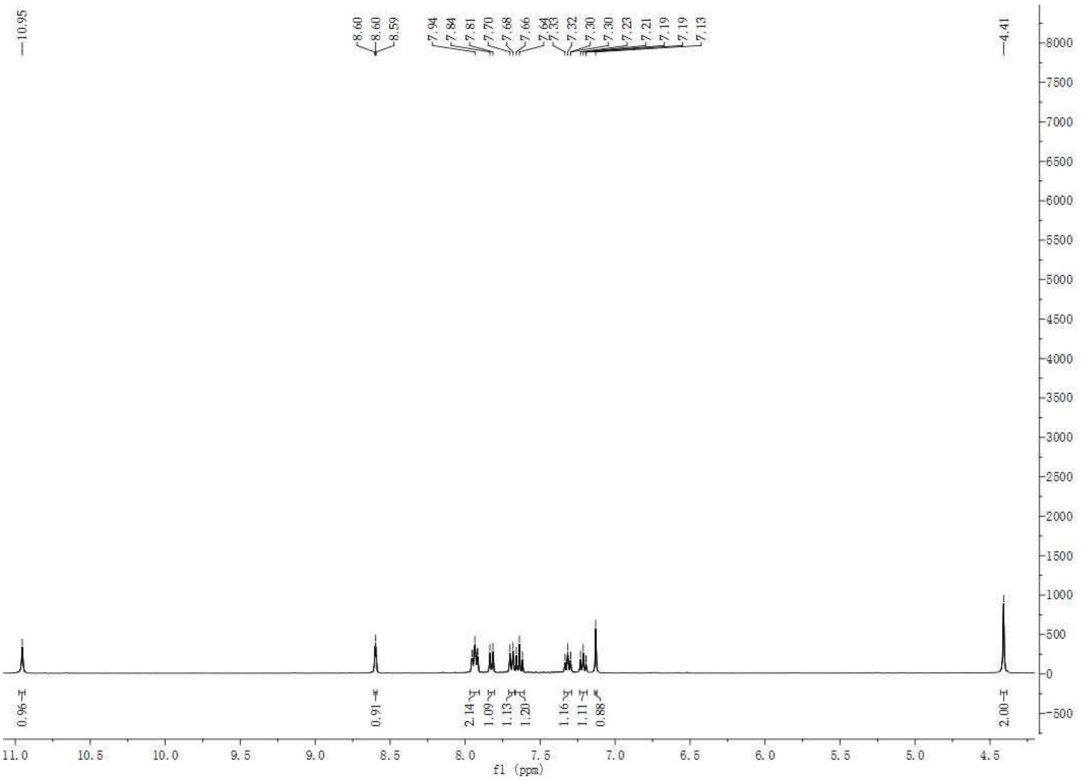

Figure S27. ^{1}H-NMR spectra of the target compound D06

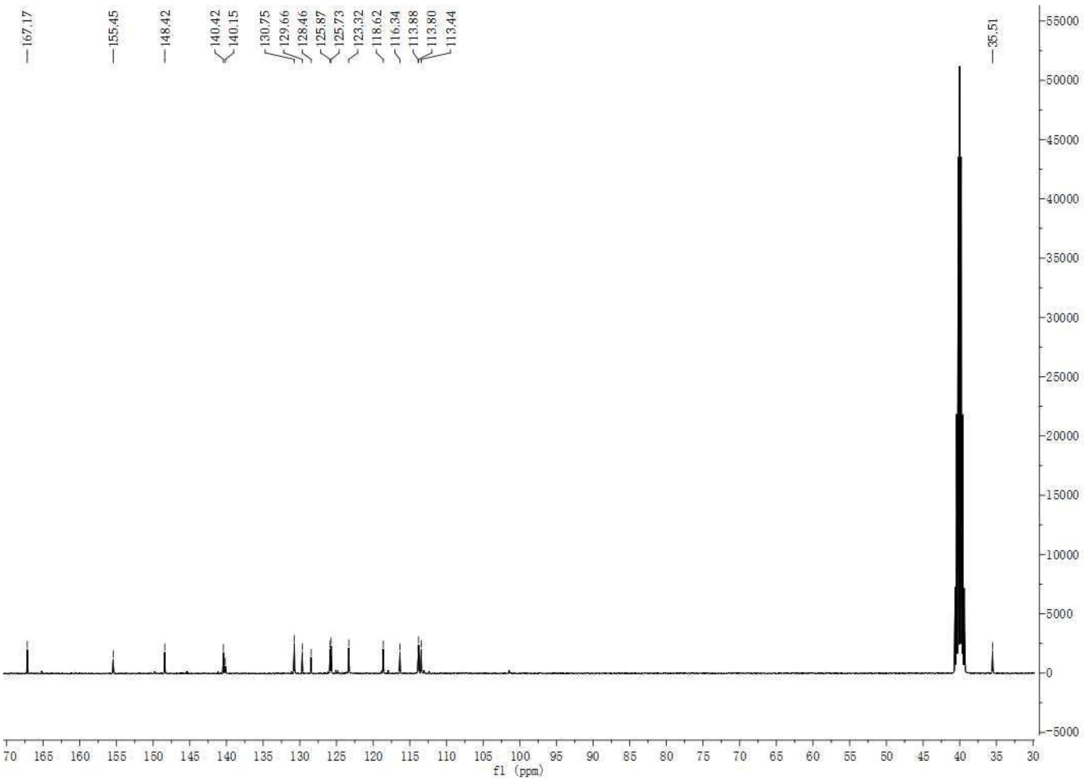

Figure S28. ^{13}C-NMR spectra of the target compound D06

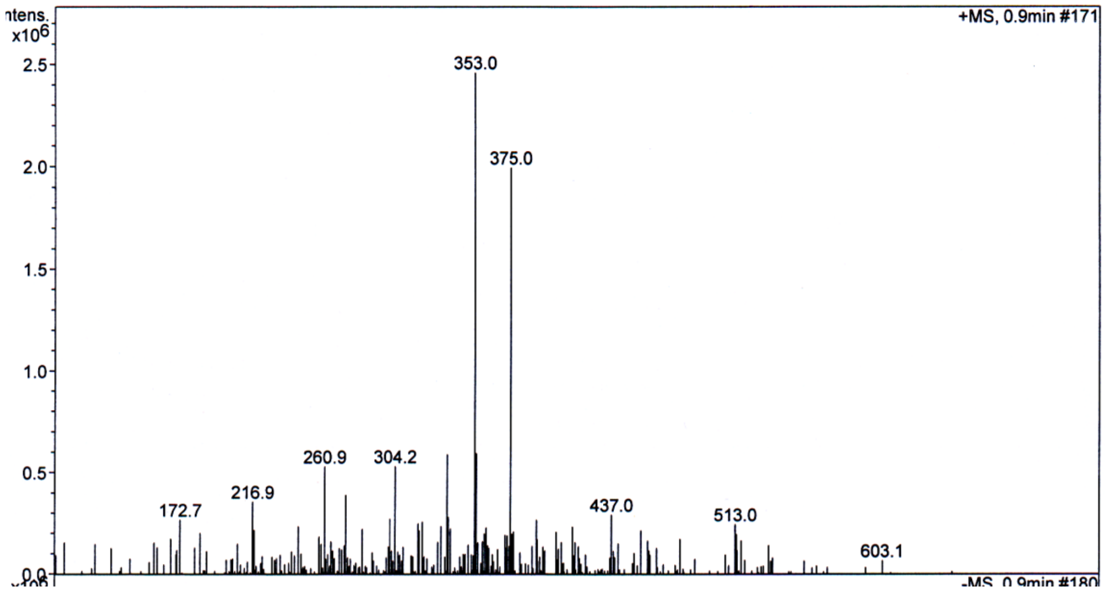

Figure S29. ESI-MS of the target compound D06

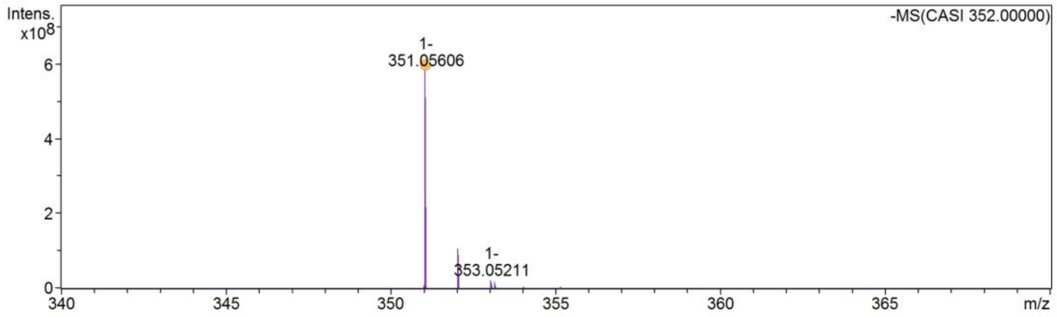

Meas. m/z	#	Ion Formula	Score	m/z	err [ppm]
351.056060	1	C17H11N4O3S	18.36	351.055735	-0.9

Figure S30. HRMS of the target compound D06

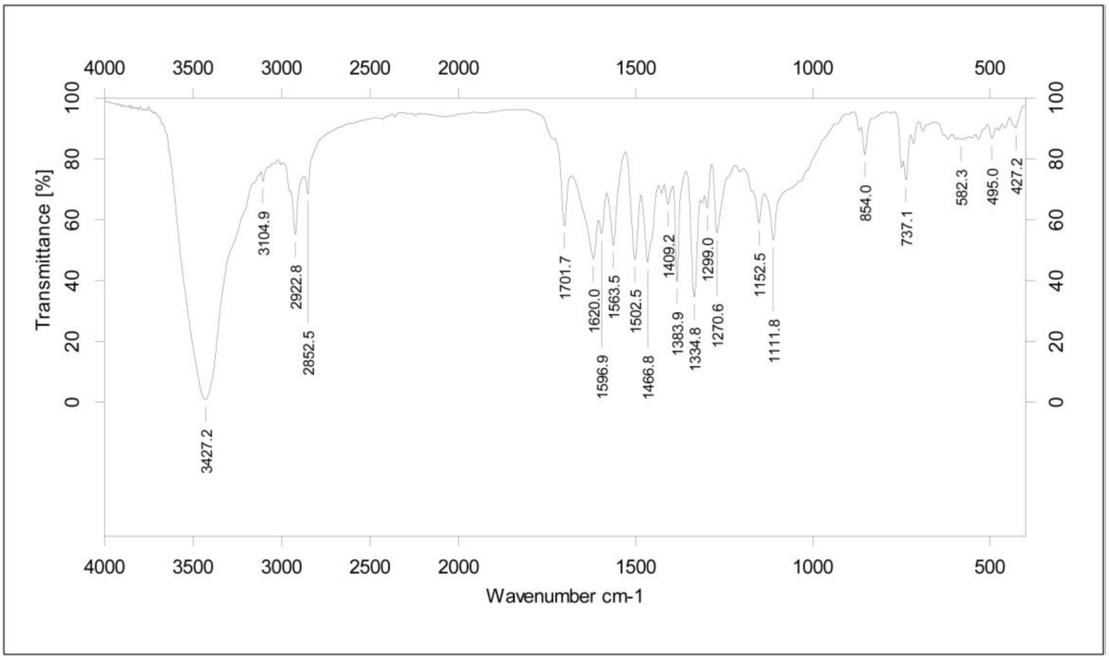

Sample : DXS-D-22	Frequency Range : 3999.64 - 400.157	Measured on : 2016-7-16	
Technique : Sample form	Resolution : 2	Instrument : EQUINOX55	Sample Scans : 16
Customer : Default	Zerofilling : 2	Acquisition : Double Sided,Forv	

Figure S31. IR spectra of the target compound D07

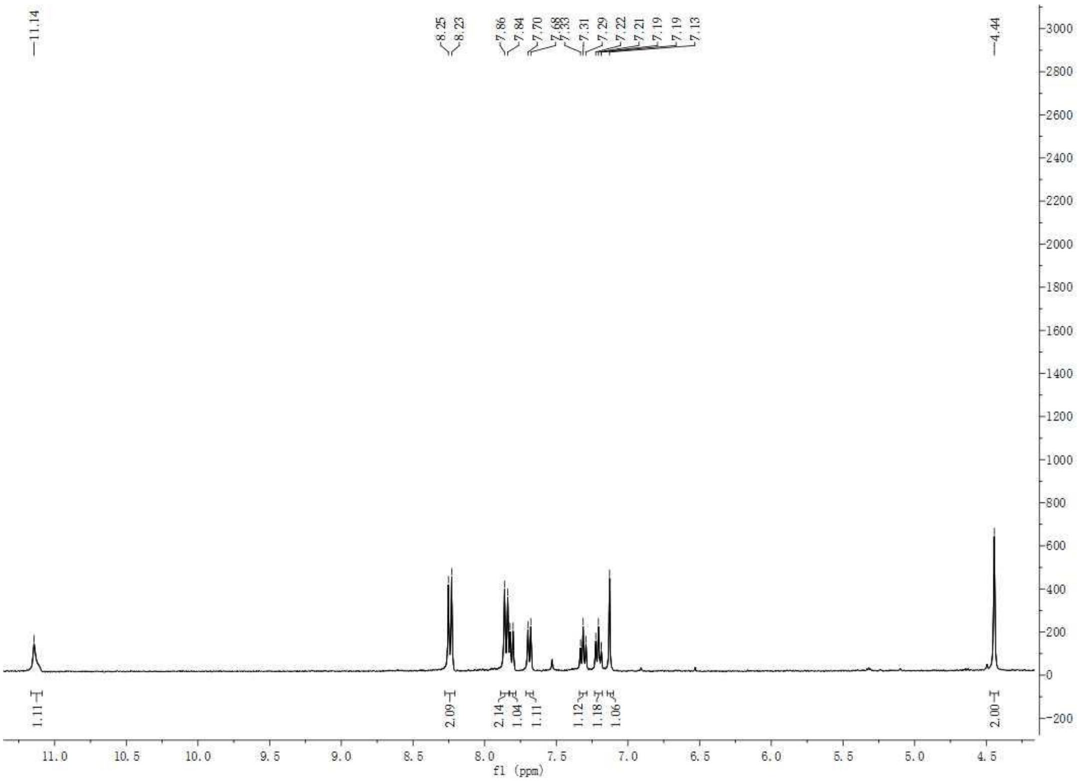

Figure S32. [1] H-NMR spectra of the target compound D07

molecules
MDPI

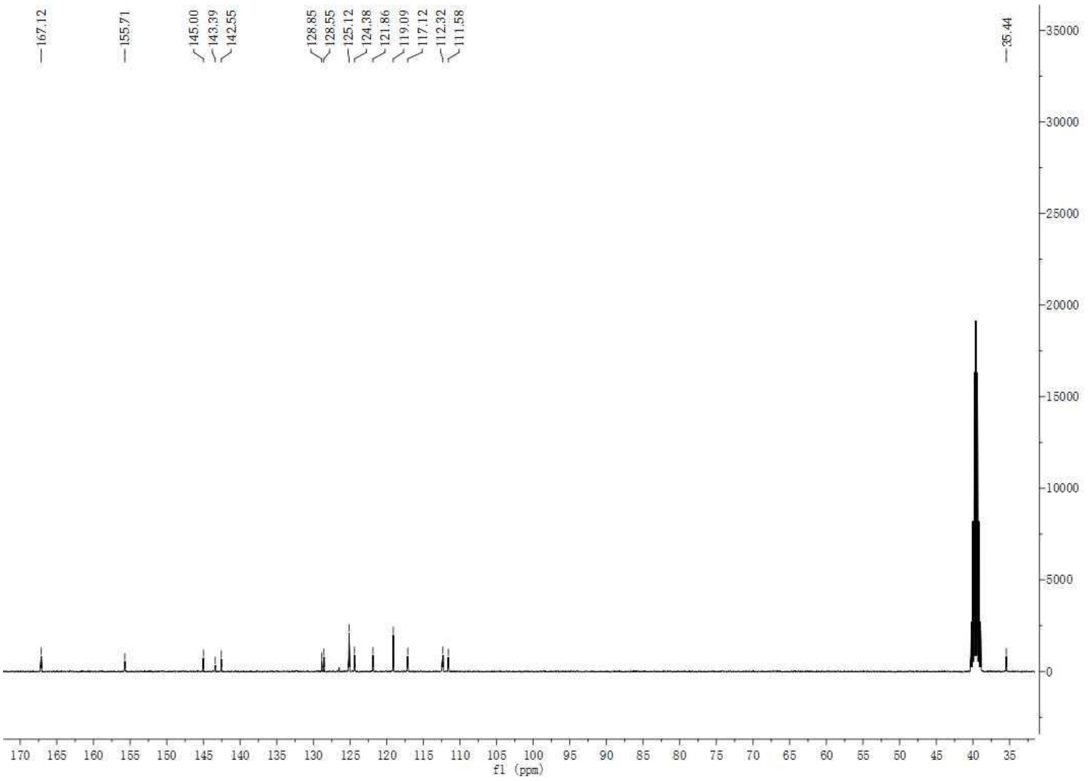

Figure S33. ^{13}C-NMR spectra of the target compound D07

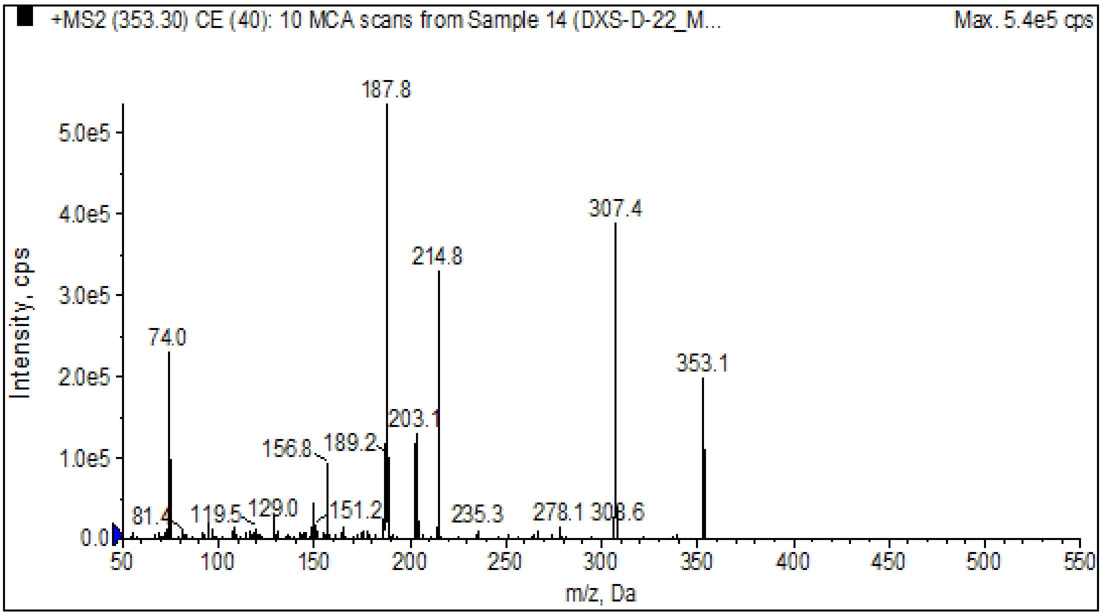

Figure S34. ESI-MS of the target compound D07

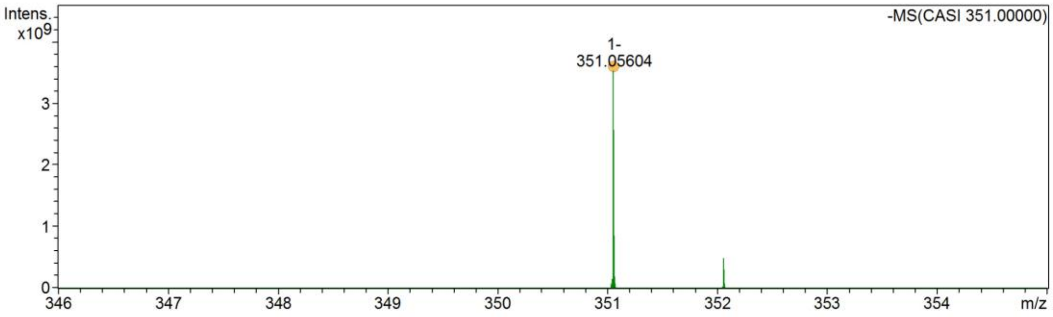

Meas. m/z	#	Ion Formula	Score	m/z	err [ppm]
351.056040	1	C17H11N4O3S	100.00	351.055735	-0.9

Figure S35. HRMS of the target compound D07

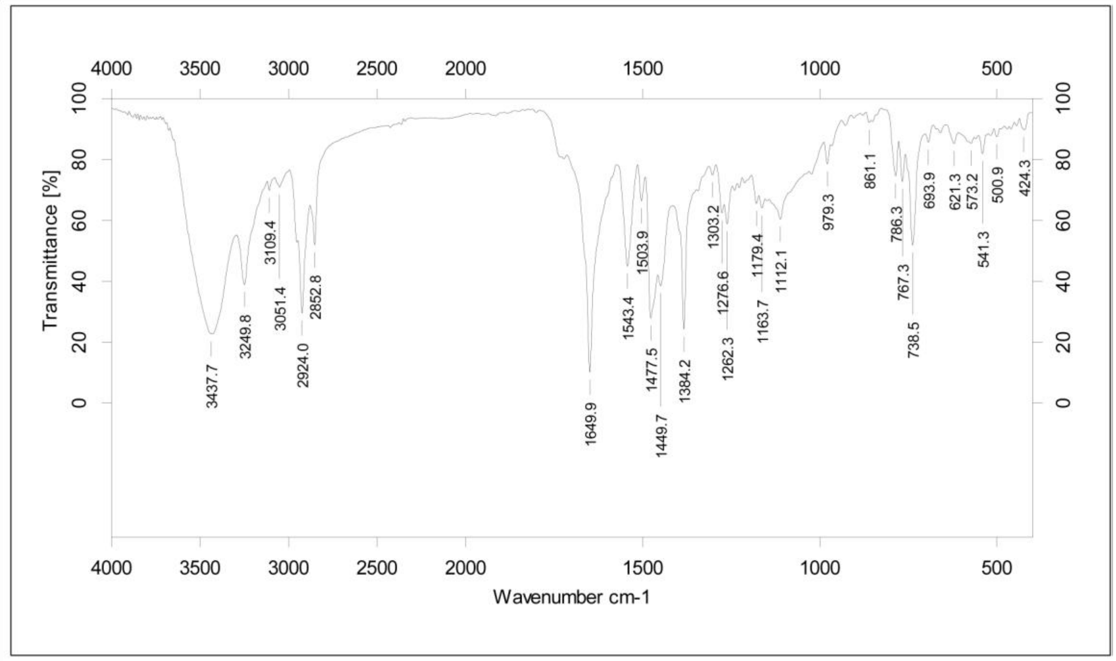

Sample : DXS-D-35	Frequency Range : 3999.64 - 400.157	Measured on : 2016-6-18	
Technique : Sample form	Resolution : 2	Instrument : EQUINOX55	Sample Scans : 16
Customer : Default	Zerofilling : 2	Acquisition : Double Sided,Forv	

Figure S36. IR spectra of the target compound D08

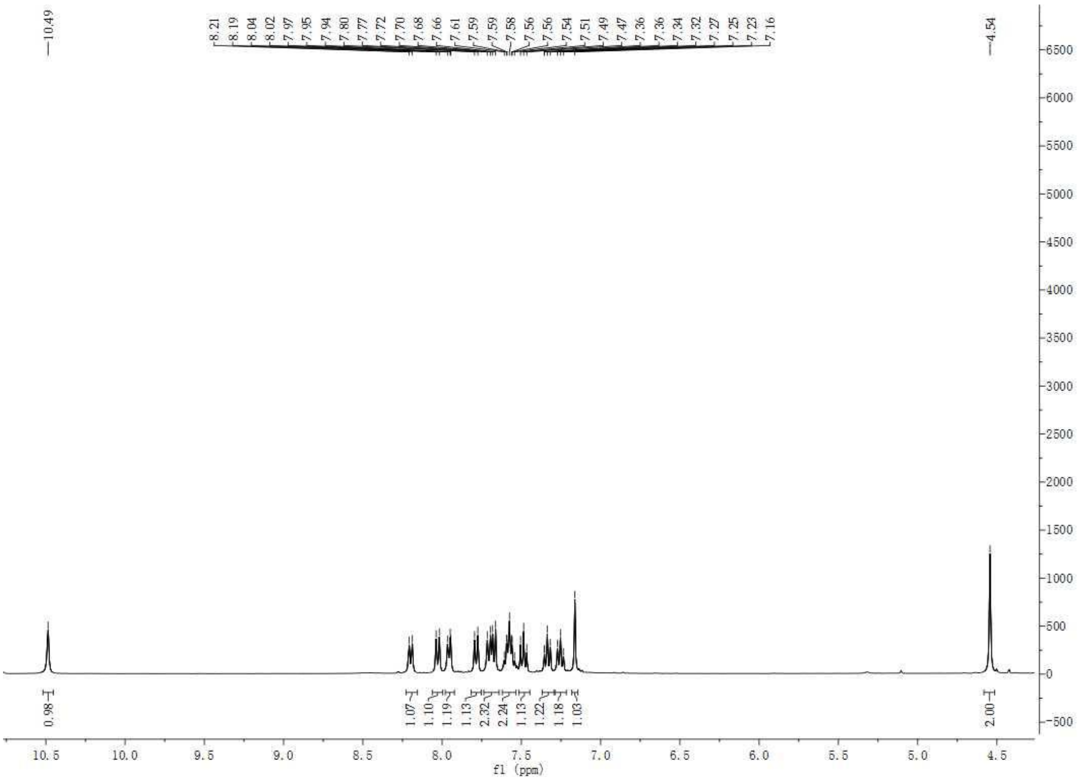

Figure S37. ^{1}H-NMR spectra of the target compound D08

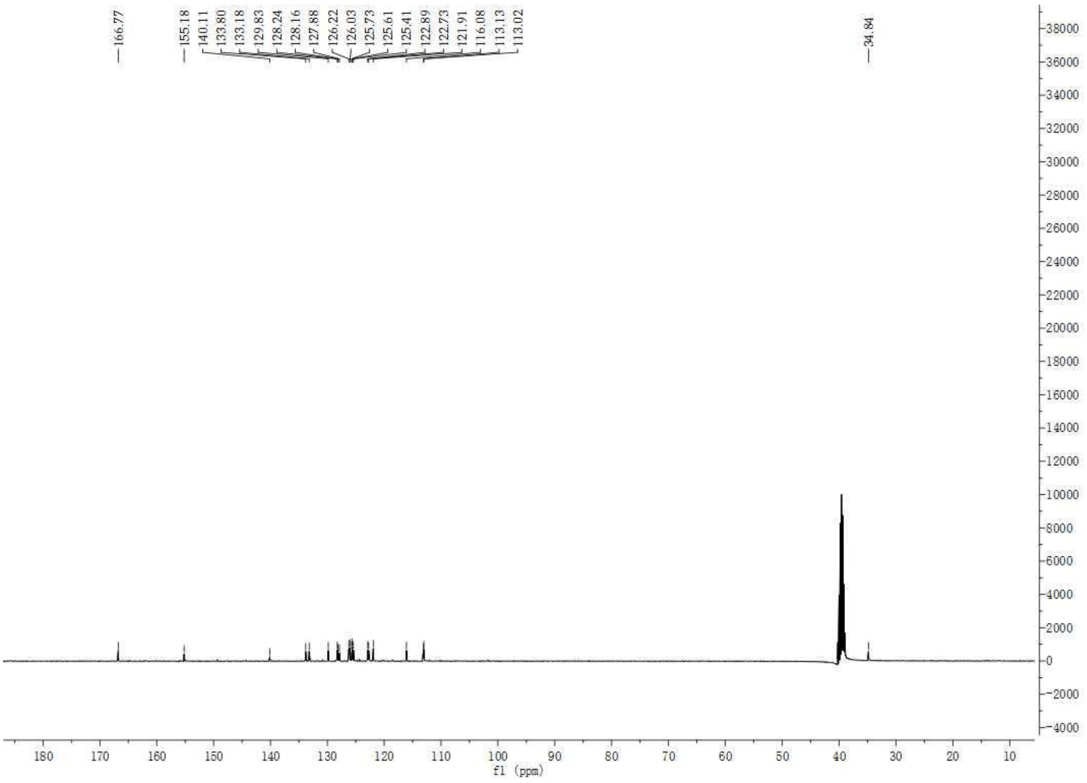

Figure S38. ^{13}C-NMR spectra of the target compound D08

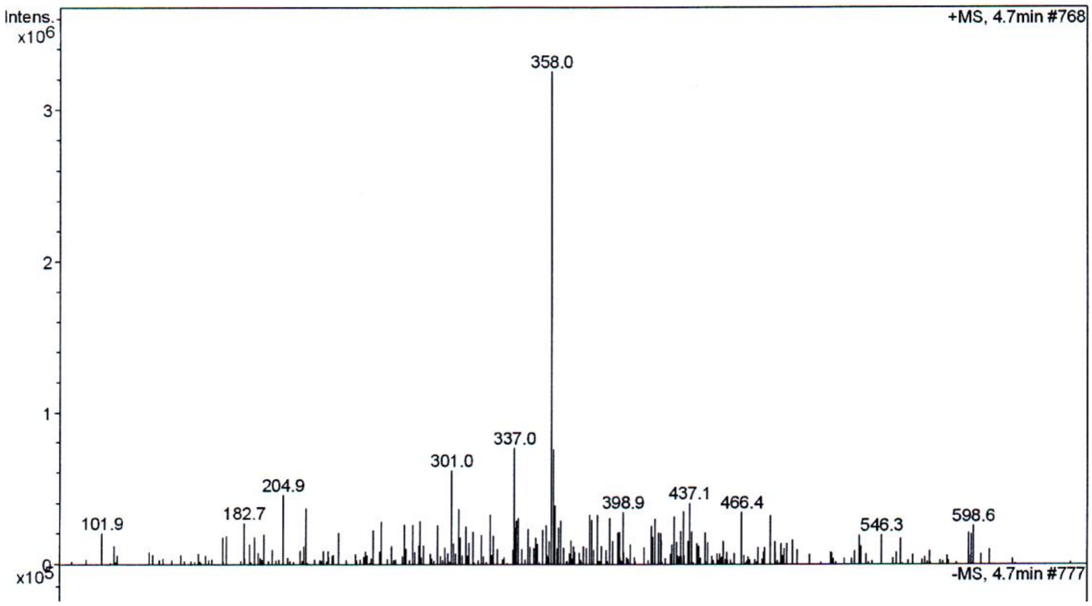

Figure S39. ESI-MS of the target compound D08

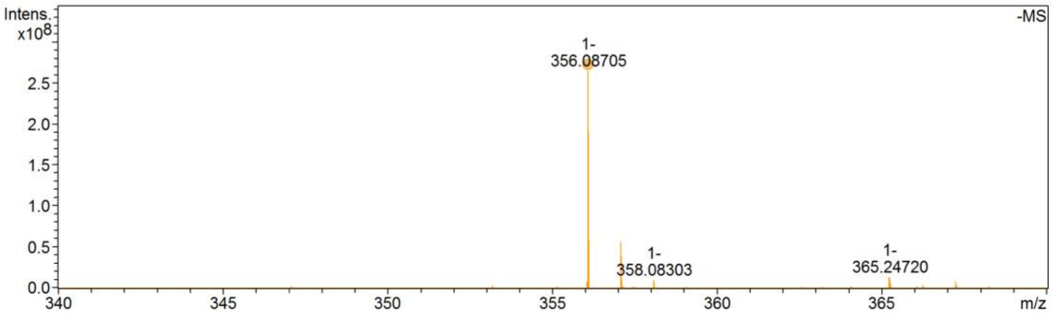

Meas. m/z	#	Ion Formula	Score	m/z	err [ppm]
356.087045	1	C21H14N3OS	100.00	356.086307	-2.1

Figure S40. HRMS of the target compound D08

molecules

MDPI

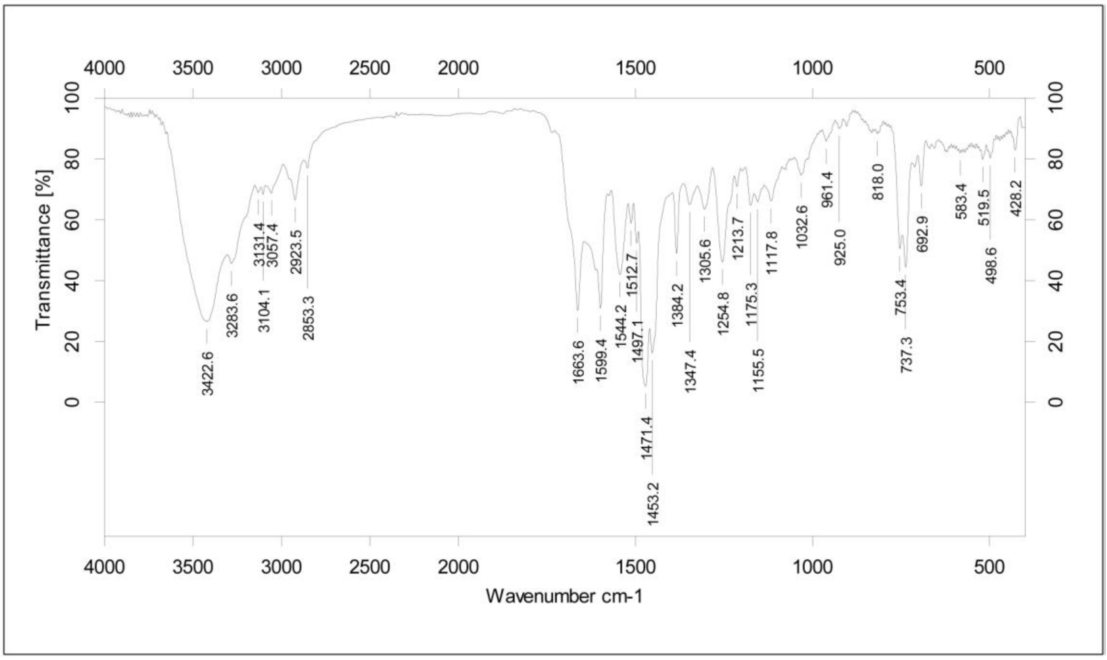

Sample : DXS-D-4		Frequency Range : 3999.64 - 400.157	Measured on : 2016-7-16
Technique : Sample form	Resolution : 2	Instrument : EQUINOX55	Sample Scans : 16
Customer : Default	Zerofilling : 2	Acquisition : Double Sided,Forv	

Figure S41. IR spectra of the target compound D09

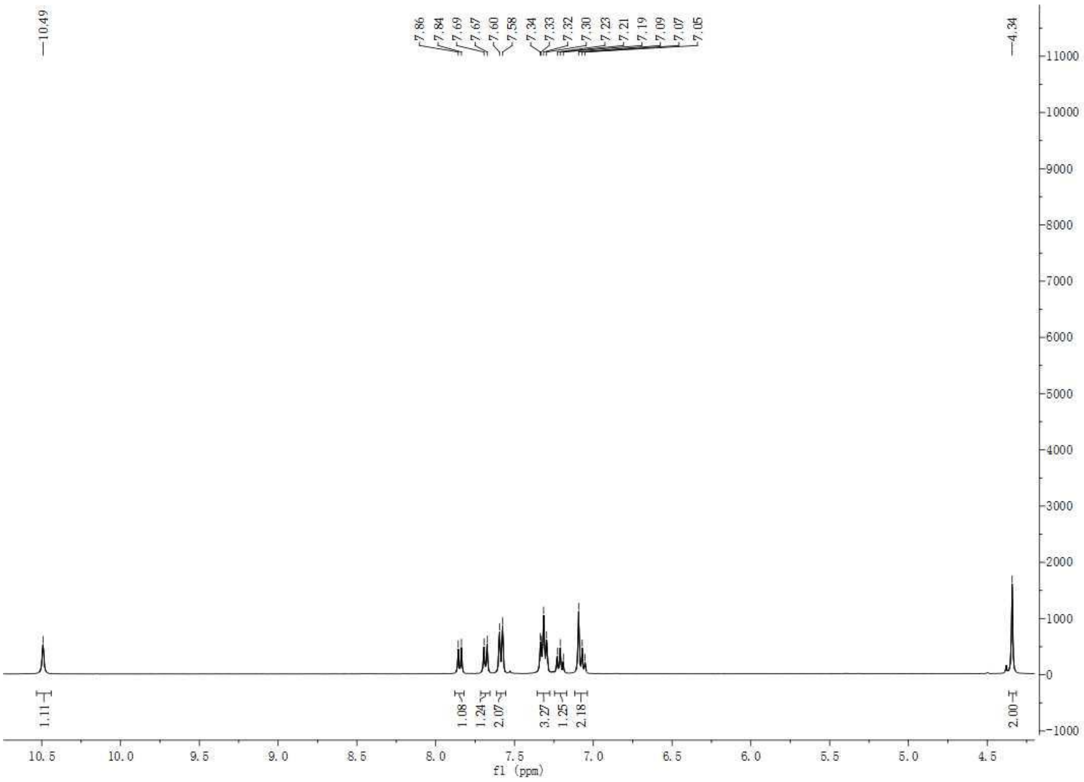

Figure S42. [1] H-NMR spectra of the target compound D09

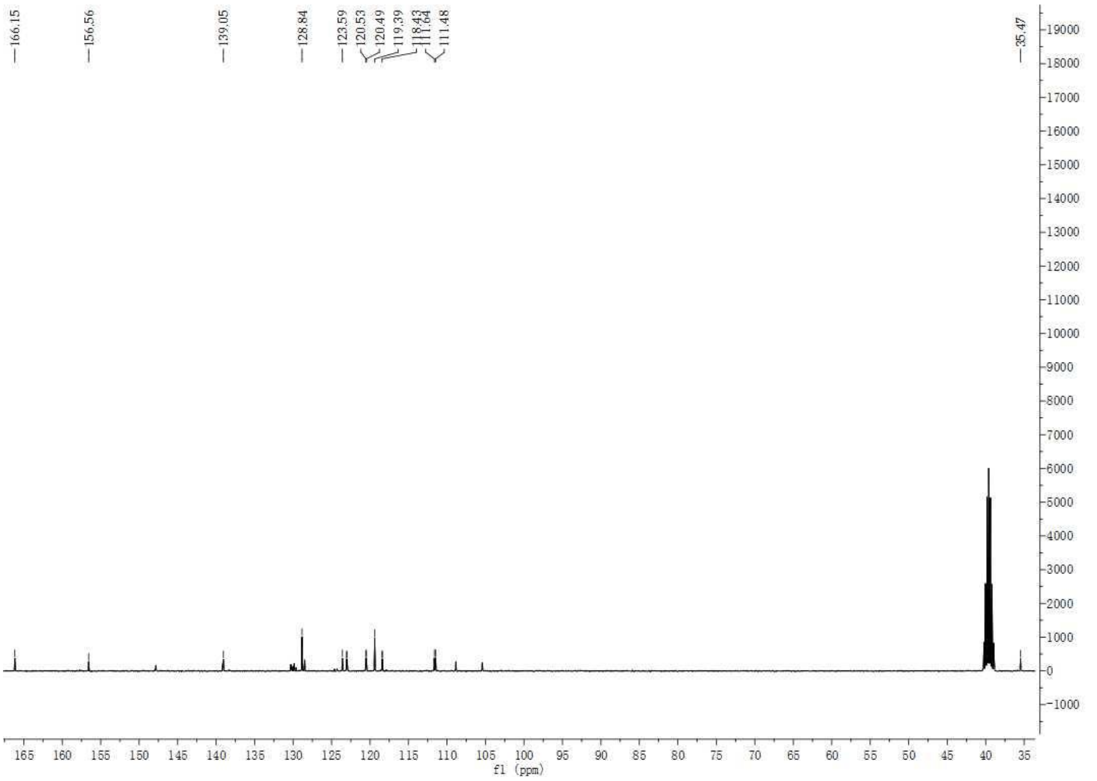

Figure S43. ^{13}C-NMR spectra of the target compound D09

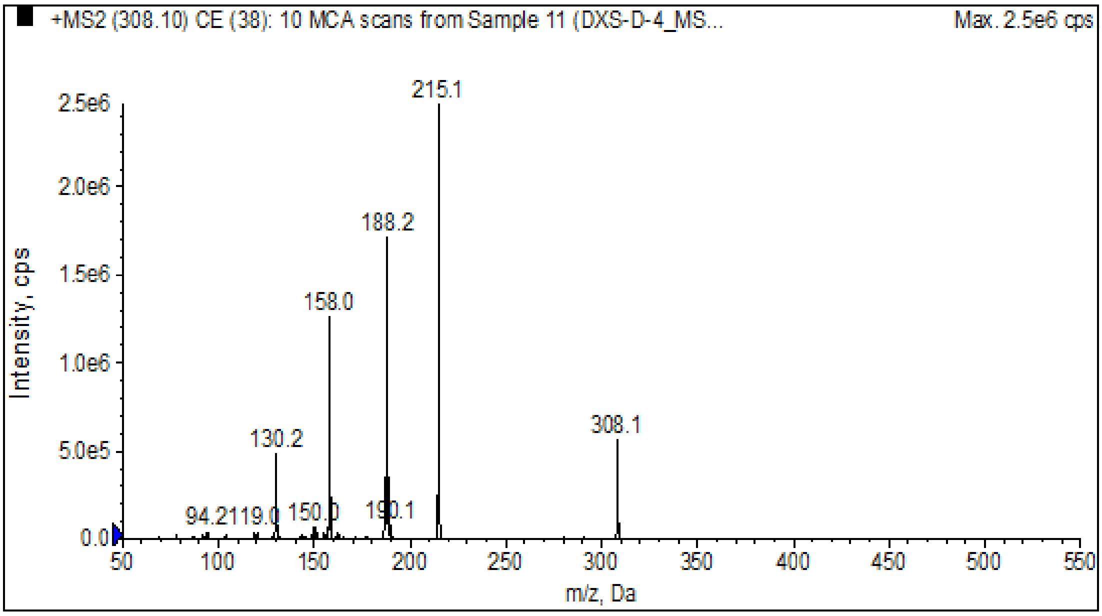

Figure S44. ESI-MS of the target compound D09

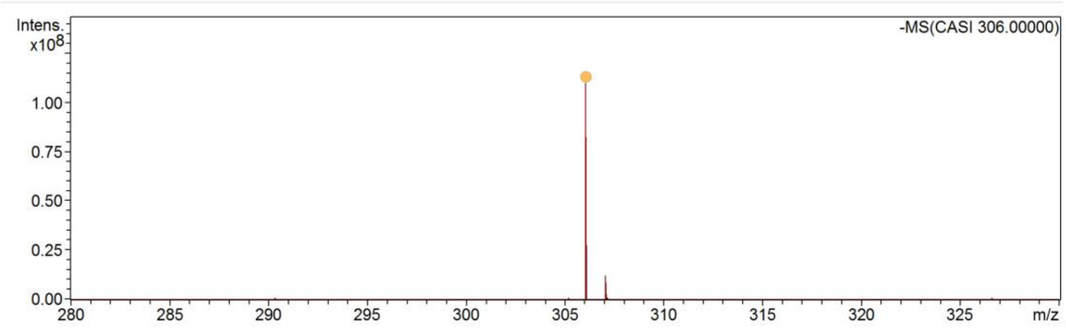

Meas. m/z	#	Ion Formula	Score	m/z	err [ppm]	Mean err [ppm]	mSigma	rdb	e⁻ Conf	N-Rule
306.070300	1	C17H12N3OS	n.a.	306.070657	1.2		n.a.	13.0	even	ok

Figure S45. HRMS of the target compound D09

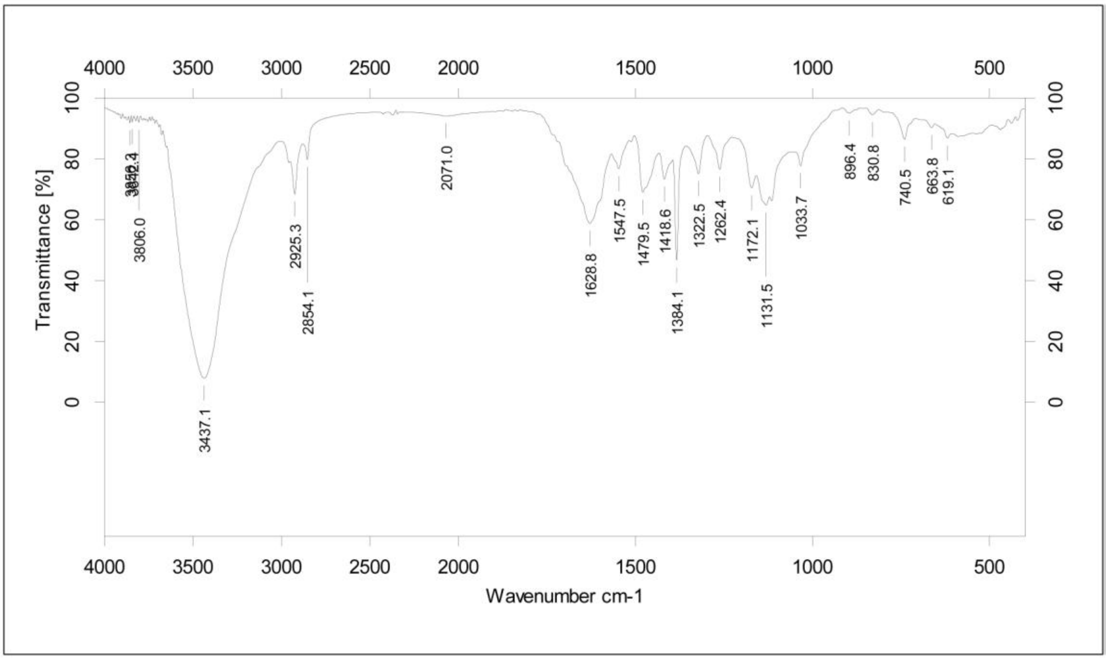

Sample : DXS-E-22	Frequency Range : 3999.64 - 400.157	Measured on : 2016-6-18	
Technique : Sample form	Resolution : 2	Instrument : EQUINOX55	Sample Scans : 16
Customer : Default	Zerofilling : 2	Acquisition : Double Sided,Forv	

Figure S46. IR spectra of the target compound D10

molecules

MDPI

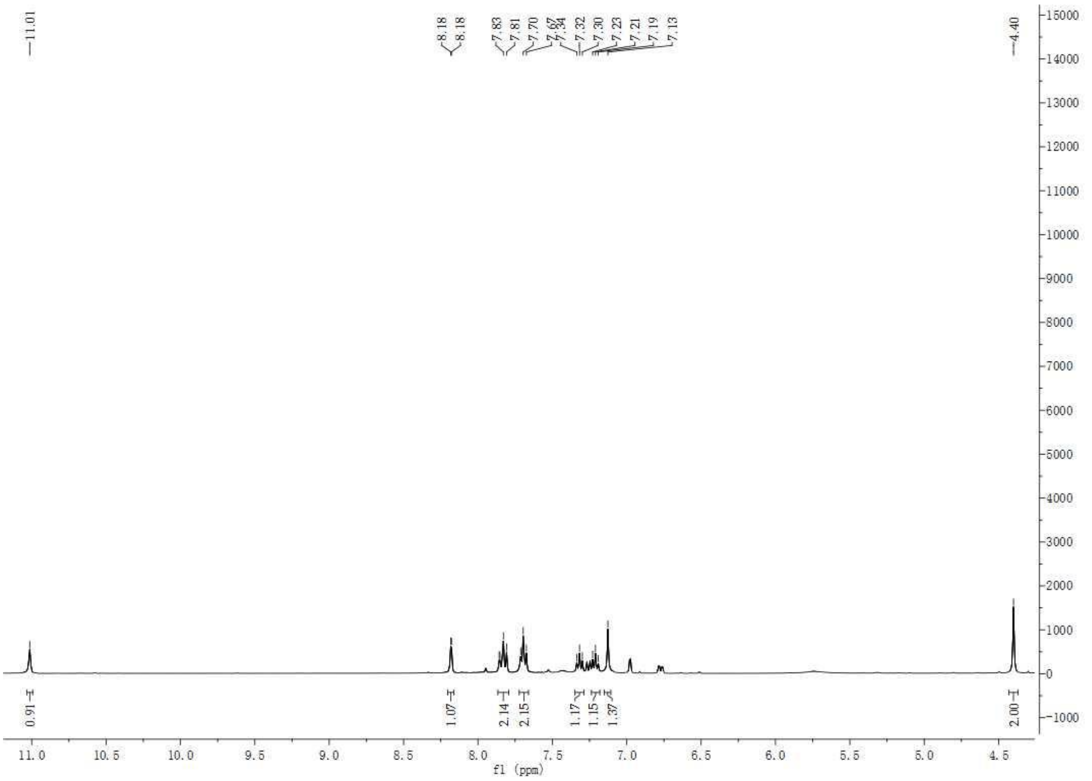

Figure S47. [1] H-NMR spectra of the target compound D10

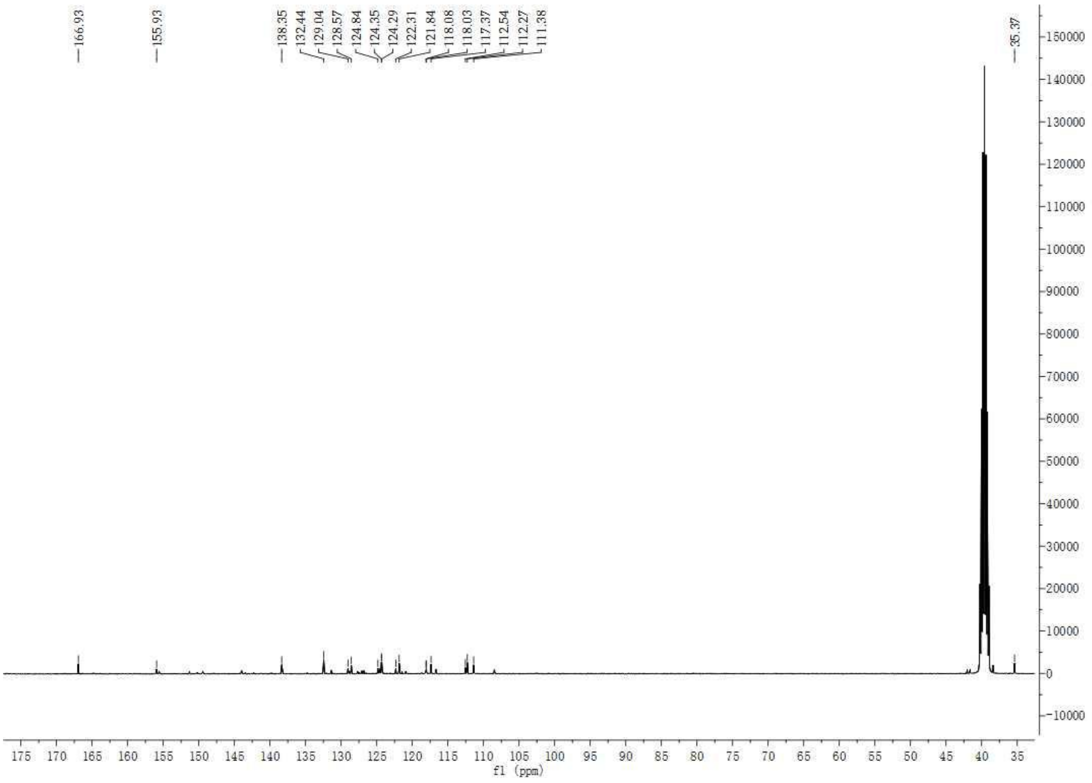

Figure S48. [13] C-NMR spectra of the target compound D10

molecules MDPI

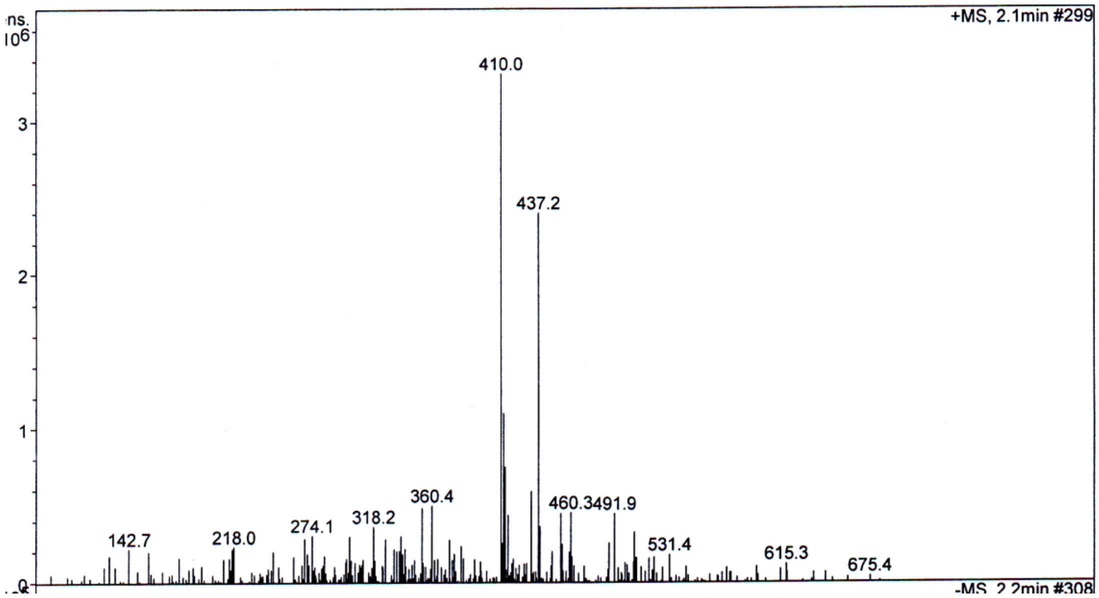

Figure S49. ESI-MS of the target compound D10

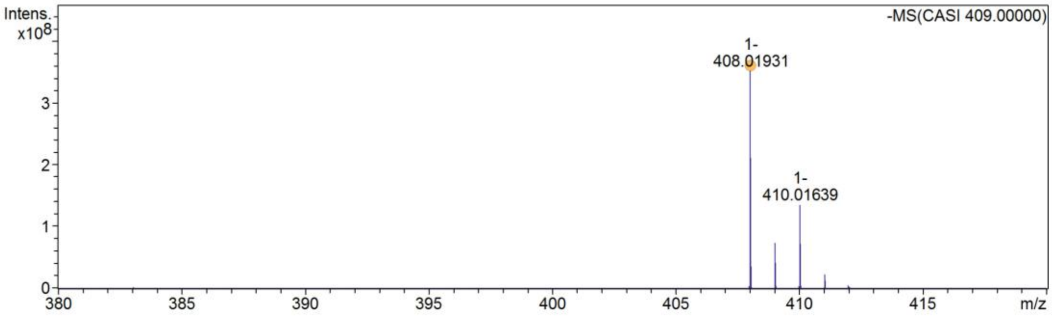

Meas. m/z	#	Ion Formula	Score	m/z	err [ppm]
408.019311	1	C18H10ClF3N3OS	28.97	408.019069	-0.6

Figure S50. HRMS of the target compound D10

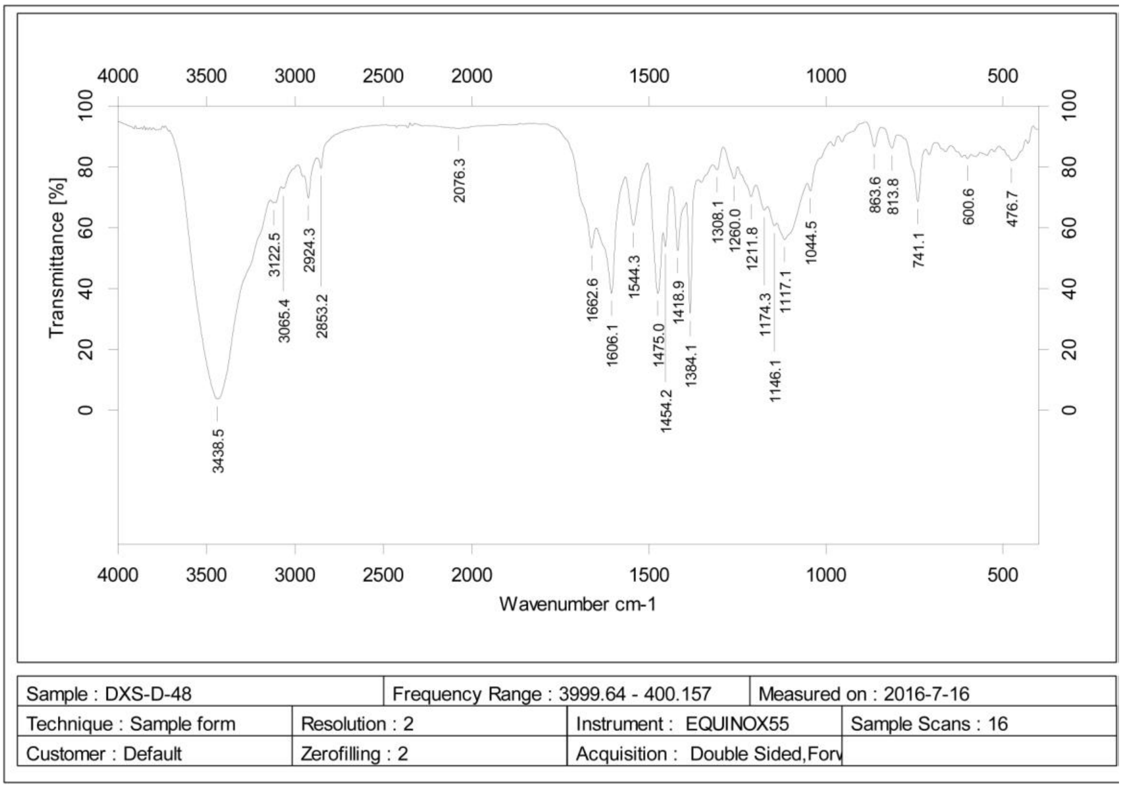

Figure S51. IR spectra of the target compound D11

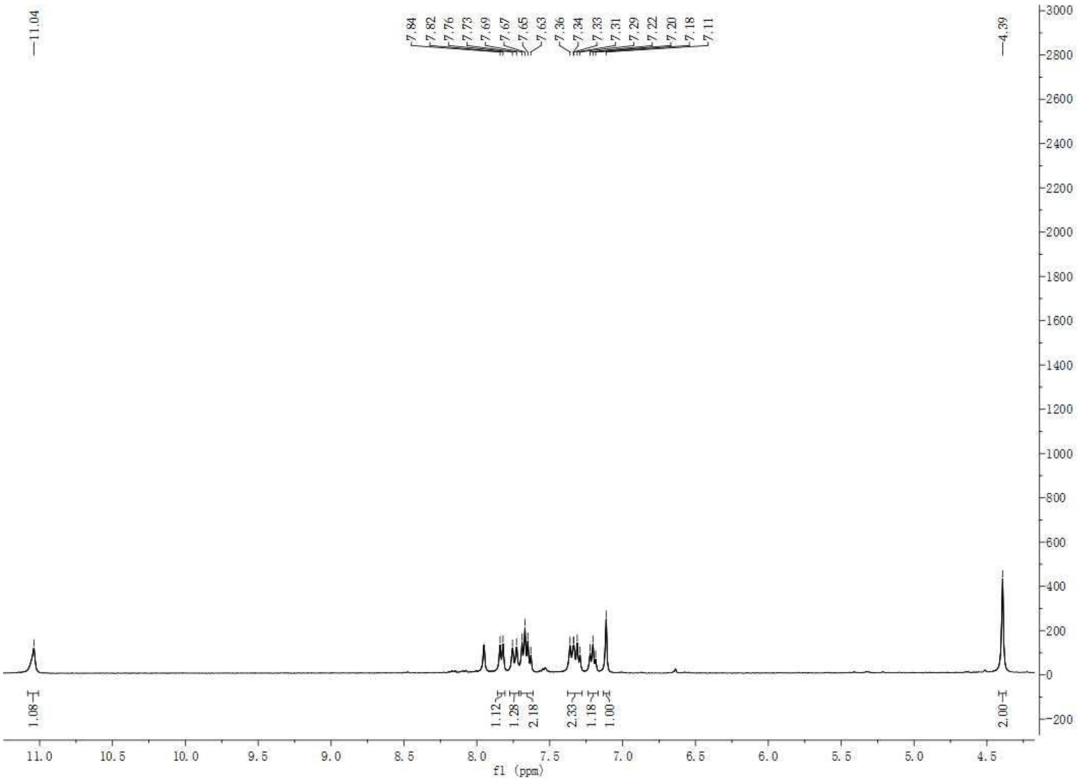

Figure S52. [1] H-NMR spectra of the target compound D11

molecules
MDPI

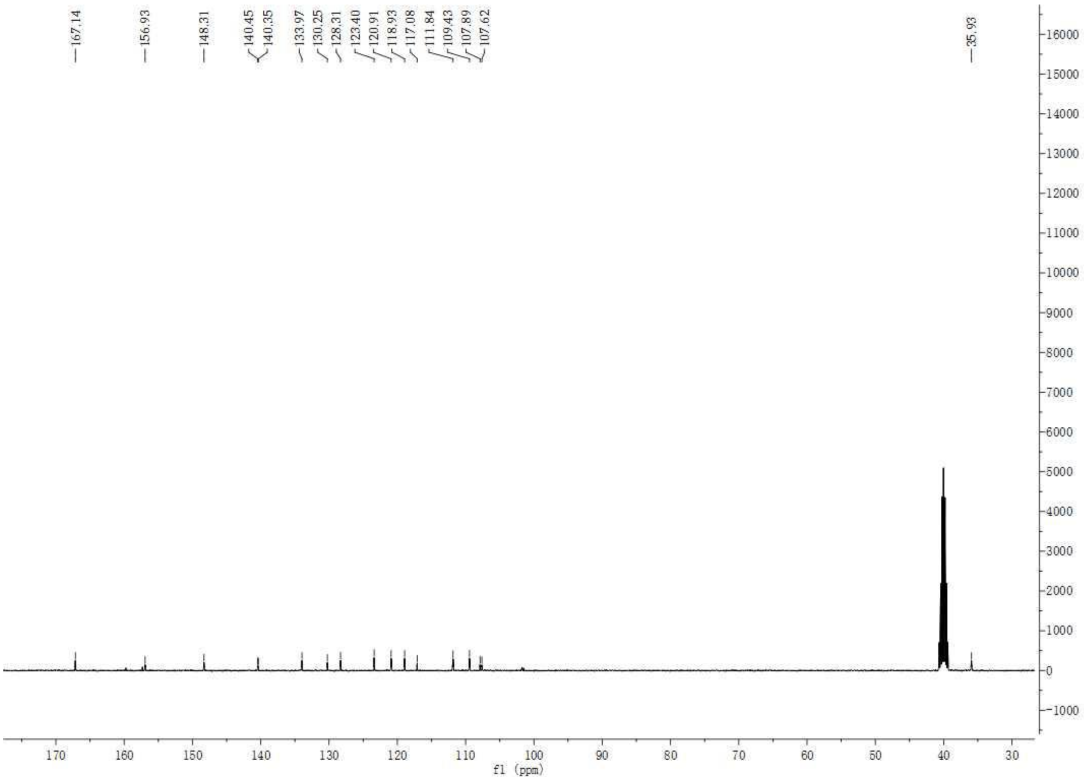

Figure S53. ^{13}C-NMR spectra of the target compound D11

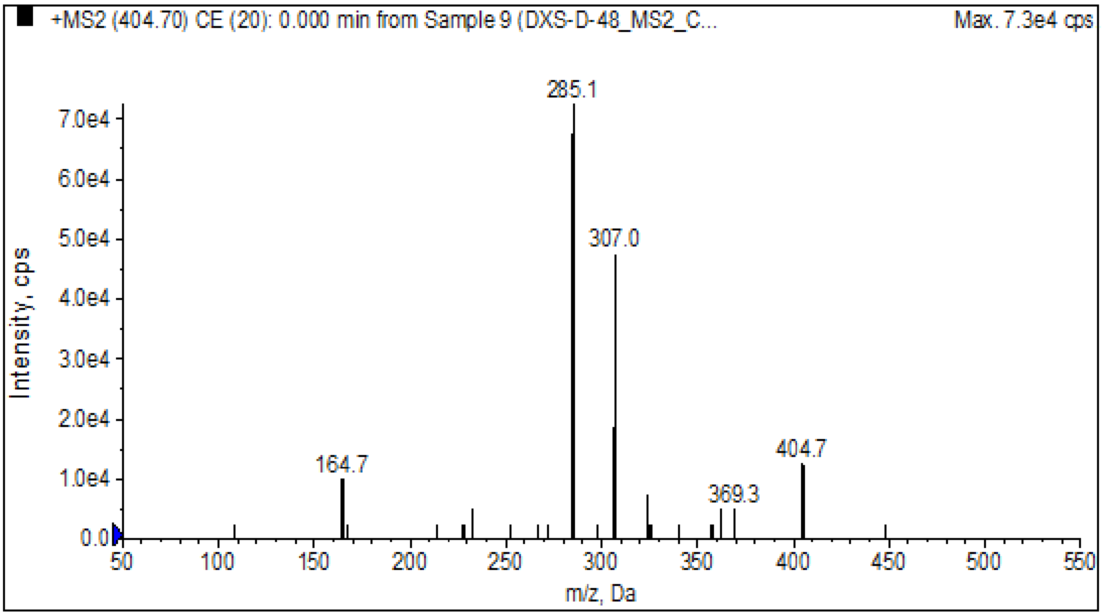

Figure S54. ESI-MS of the target compound D11

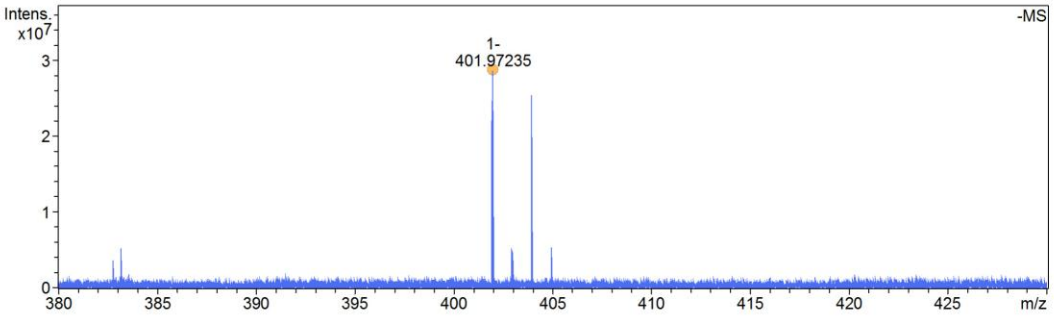

Meas. m/z	#	Ion Formula	Score	m/z	err [ppm]
401.972353	1	C17H10BrFN3OS	42.64	401.971747	-1.5

Figure S55. HRMS of the target compound D11

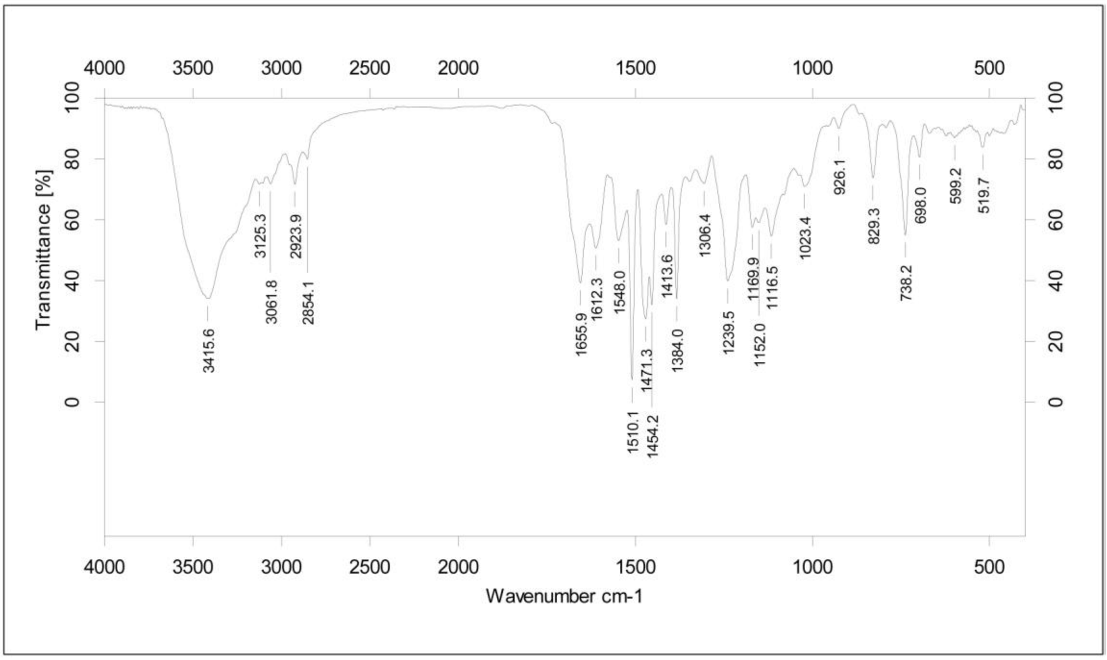

Sample : DXS-D-5	Frequency Range : 3999.64 - 400.157	Measured on : 2016-7-16	
Technique : Sample form	Resolution : 2	Instrument : EQUINOX55	Sample Scans : 16
Customer : Default	Zerofilling : 2	Acquisition : Double Sided,Forv	

Figure S56. IR spectra of the target compound D12

molecules

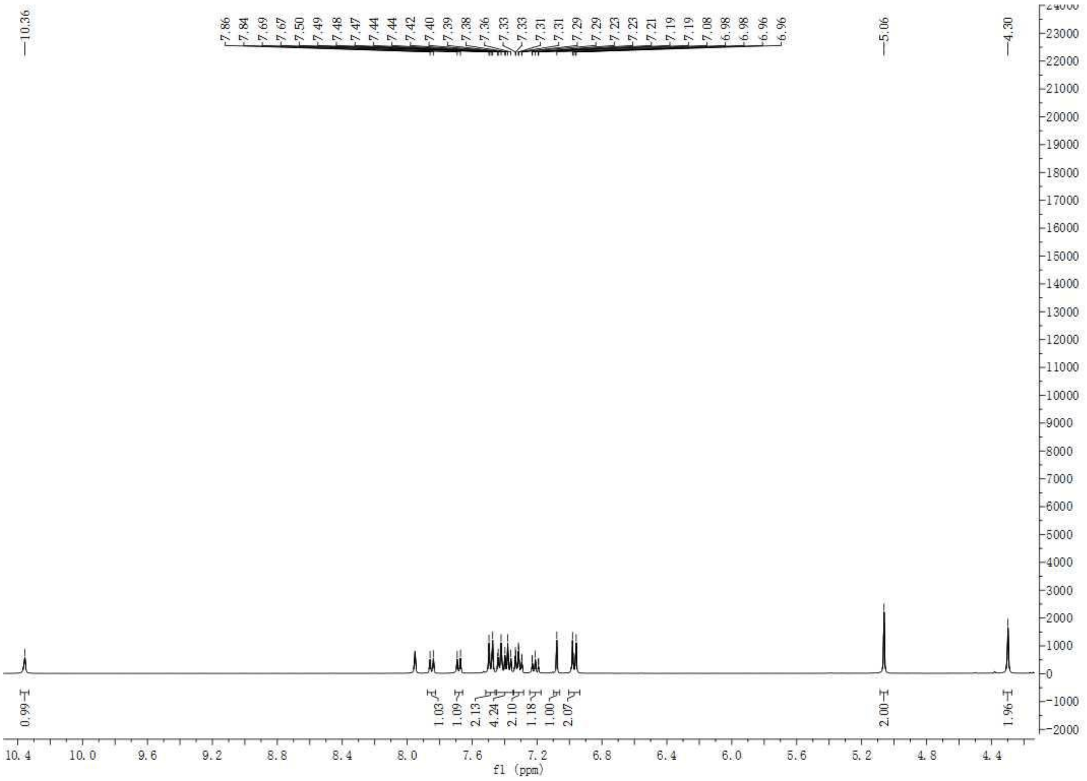

Figure S57. [1] H-NMR spectra of the target compound D12

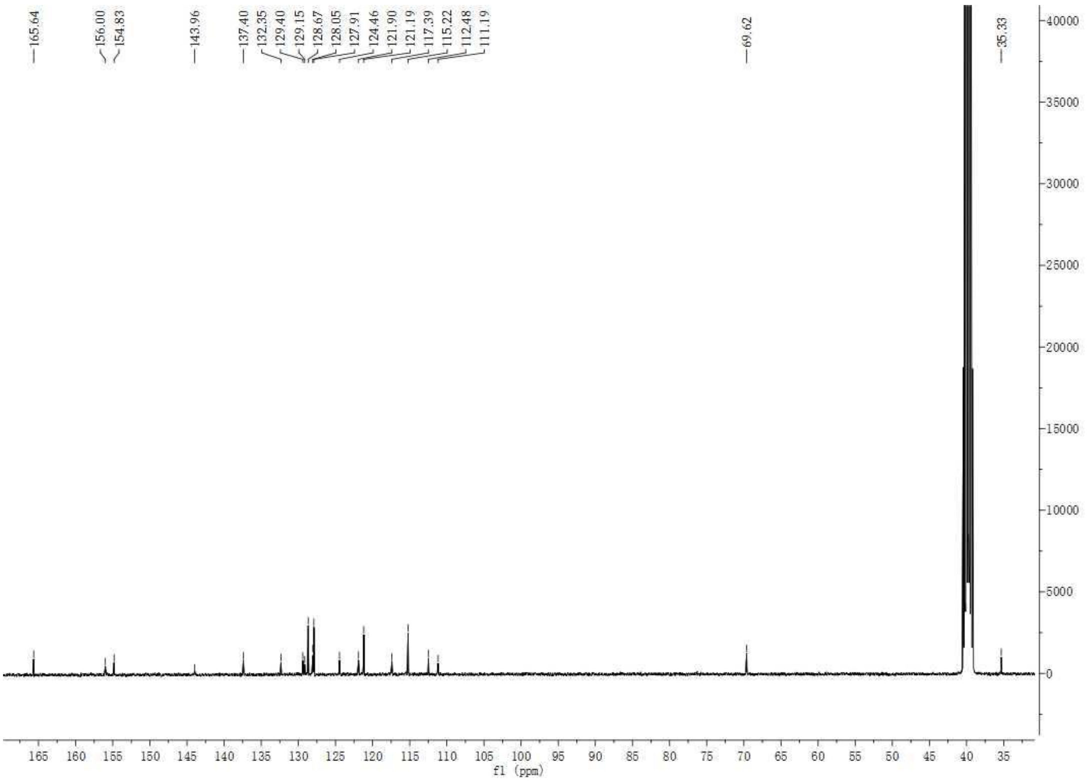

Figure S58. [13] C-NMR spectra of the target compound D12

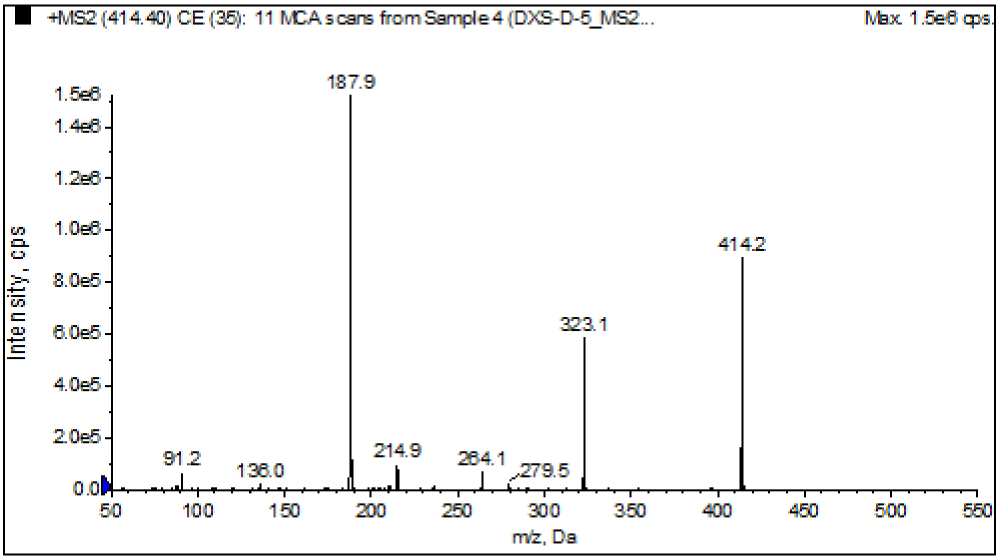

Figure S59. ESI-MS of the target compound D12

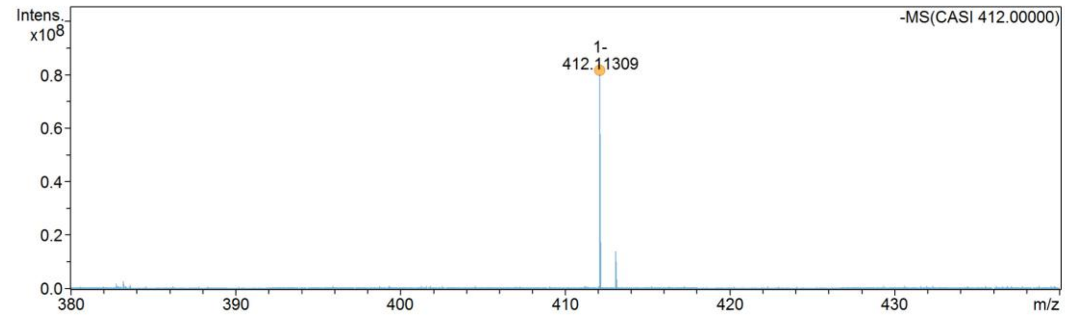

Meas. m/z	#	Ion Formula	Score	m/z	err [ppm]
412.113094	1	C24H18N3O2S	100.00	412.112521	-1.4

Figure S60. HRMS of the target compound D12

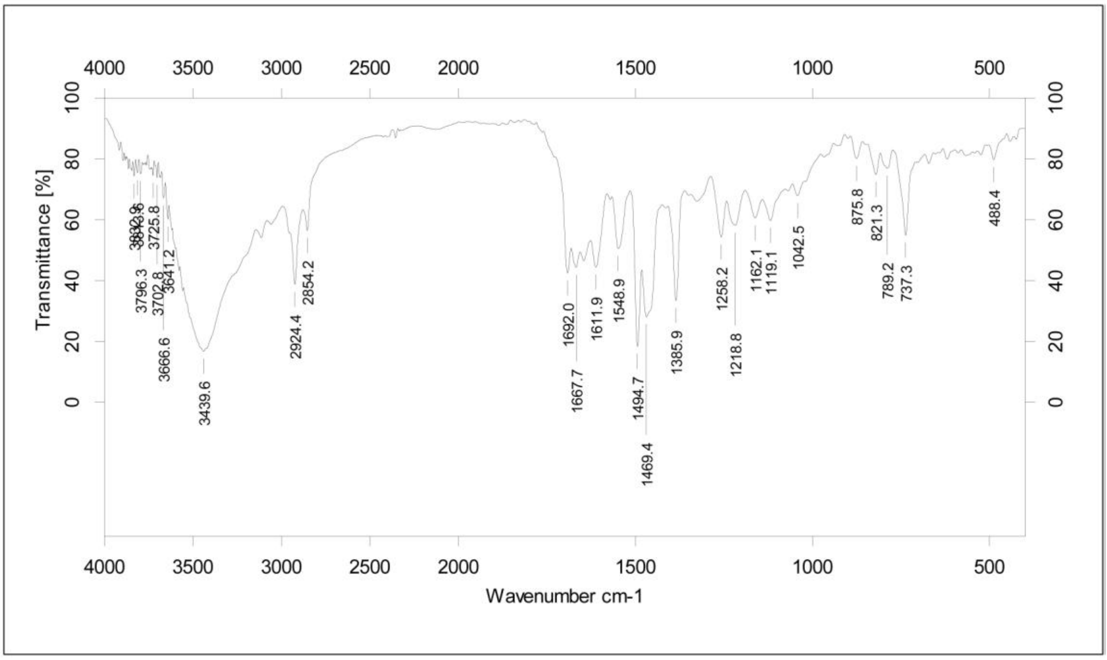

Sample : DXS-D-50		Frequency Range : 3999.64 - 400.157	Measured on : 2016-6-18
Technique : Sample form	Resolution : 2	Instrument : EQUINOX55	Sample Scans : 16
Customer : Default	Zerofilling : 2	Acquisition : Double Sided,Forv	

Figure S61. IR spectra of the target compound D13

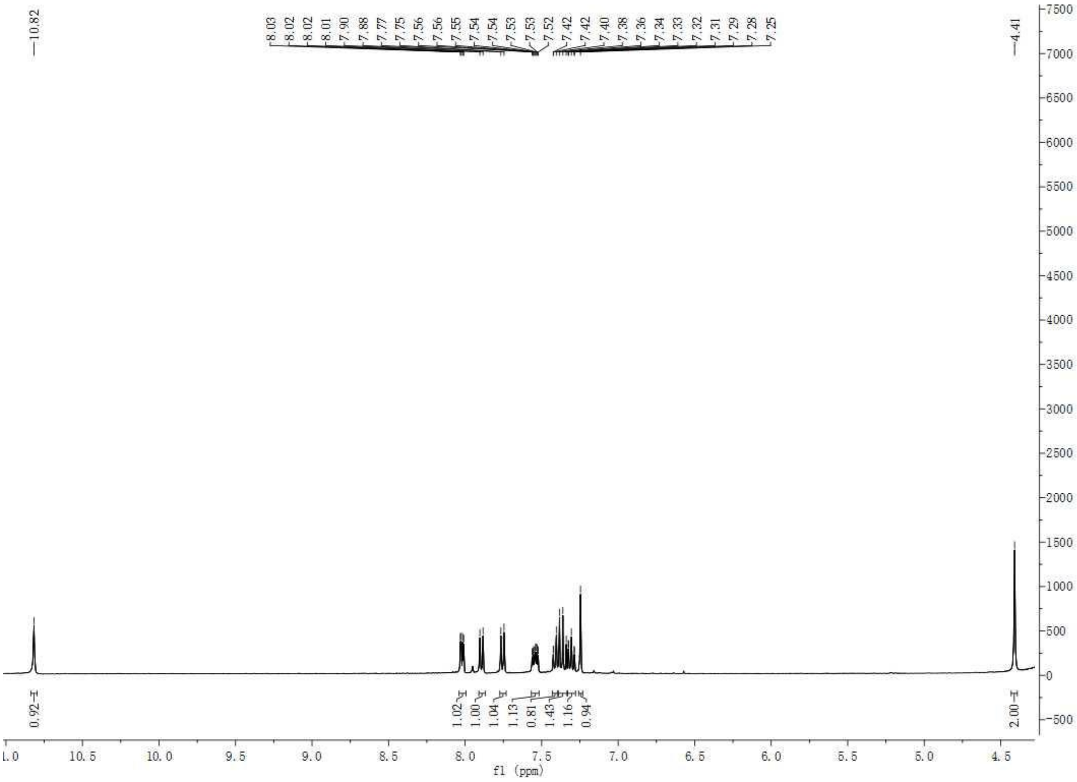

Figure S62. ^{1}H-NMR spectra of the target compound D13

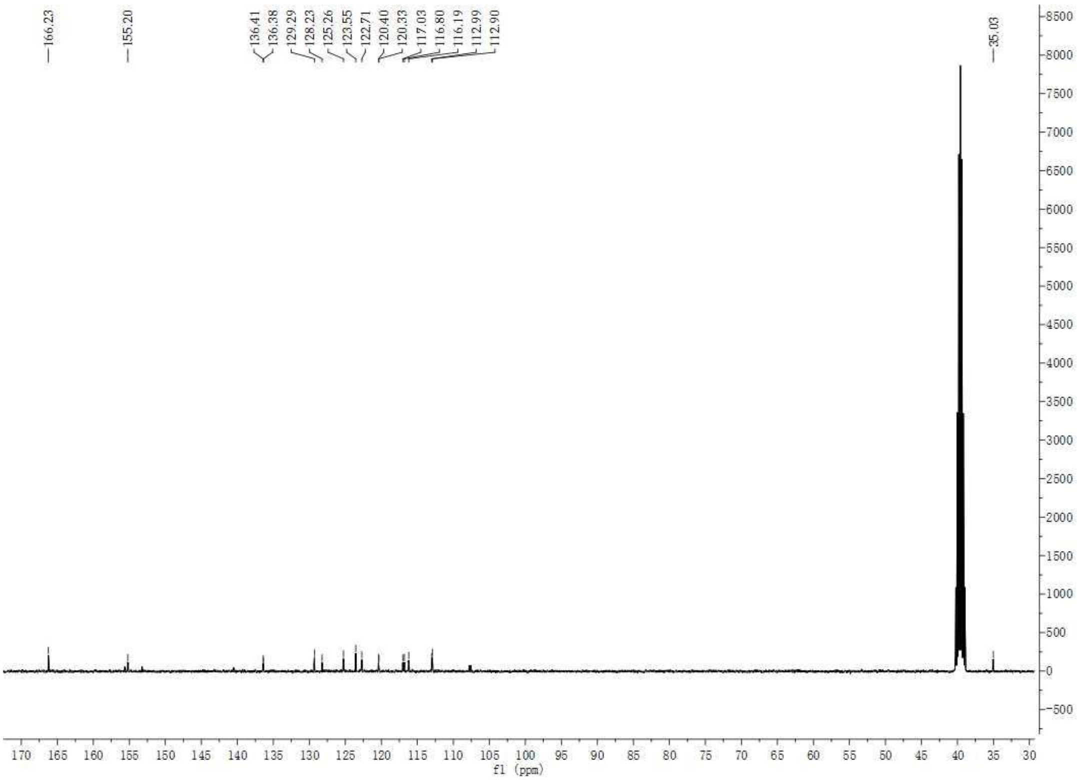

Figure S63. ^{13}C-NMR spectra of the target compound D13

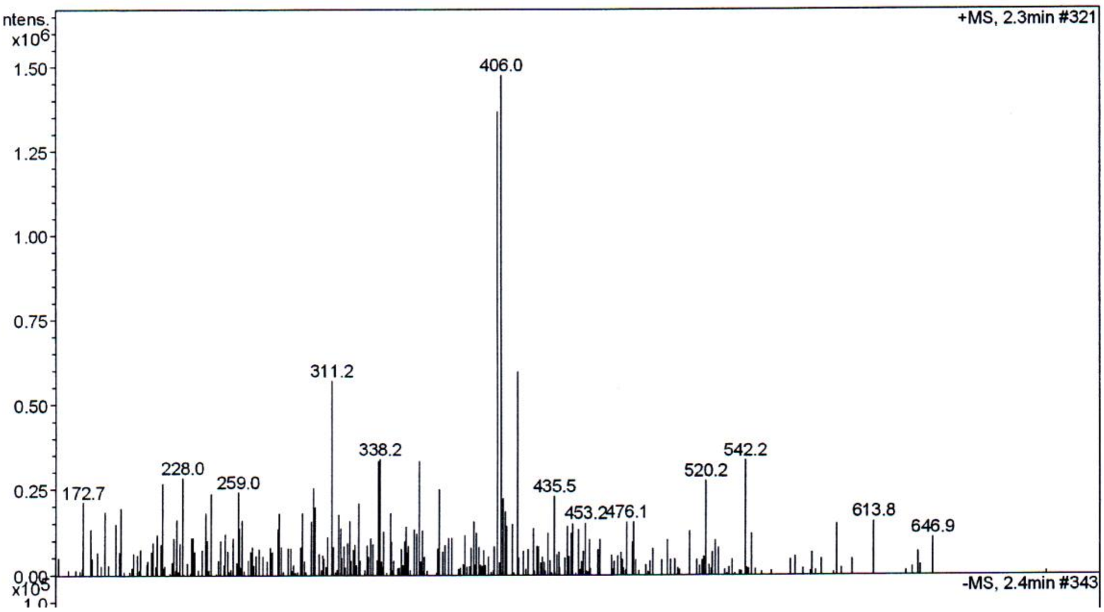

Figure S64. ESI-MS of the target compound D13

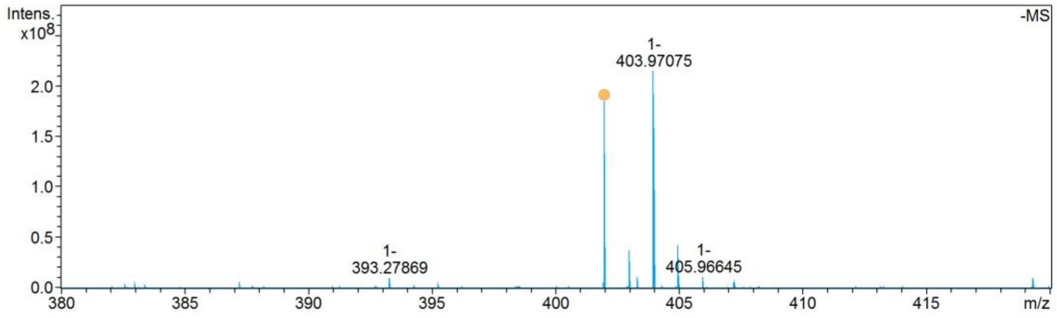

Meas. m/z	#	Ion Formula	Score	m/z	err [ppm]
401.971380	1	C17H10BrFN3OS	n.a.	401.971747	0.9

Figure S65. HRMS of the target compound D13

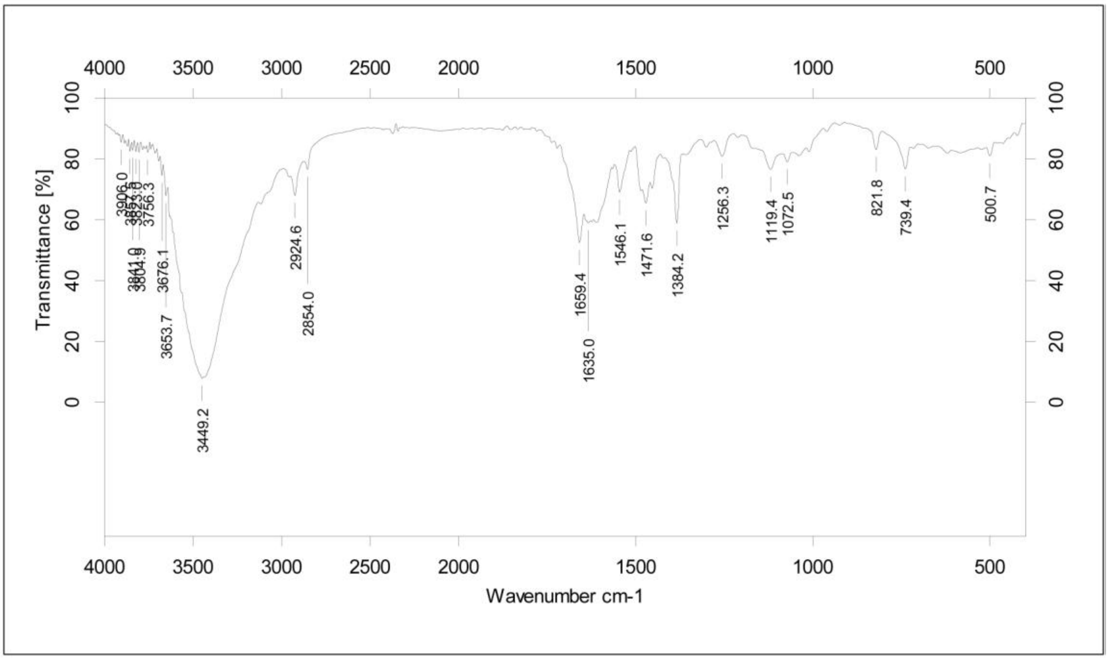

Sample : DXS-D-7	Frequency Range : 3999.64 - 400.157	Measured on : 2016-6-18	
Technique : Sample form	Resolution : 2	Instrument : EQUINOX55	Sample Scans : 16
Customer : Default	Zerofilling : 2	Acquisition : Double Sided,Forv	

Figure S66. IR spectra of the target compound D14

molecules

MDPI

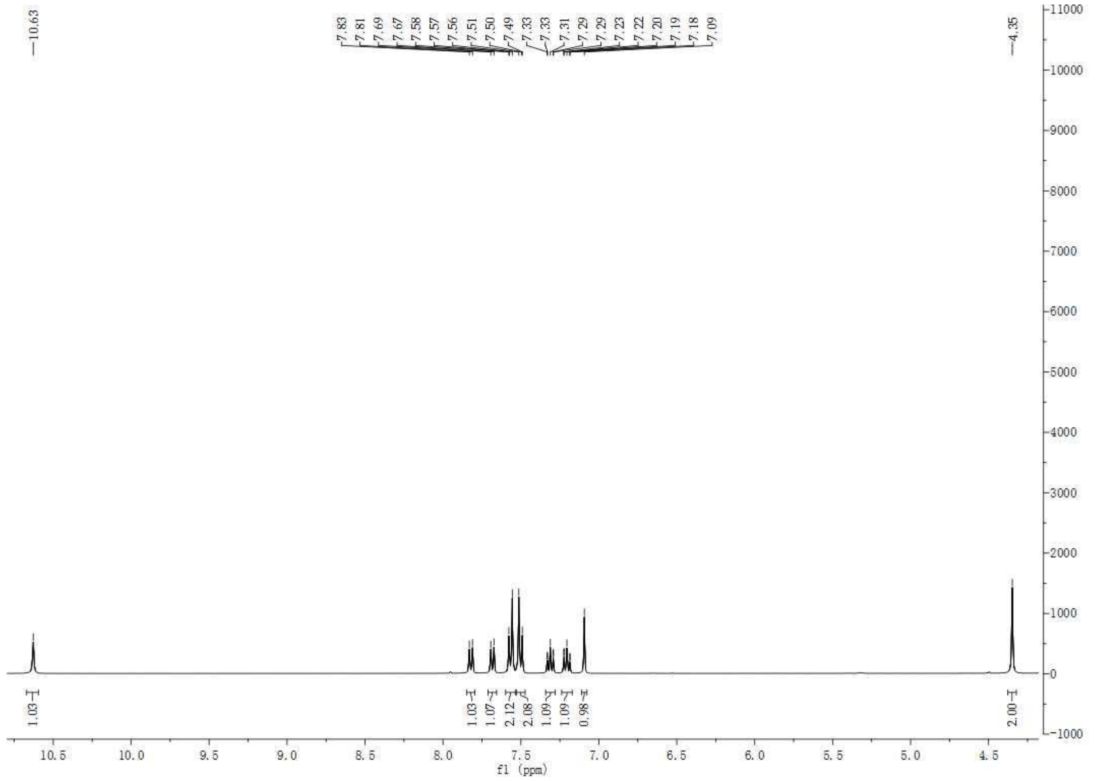

Figure S67. [1] H-NMR spectra of the target compound D14

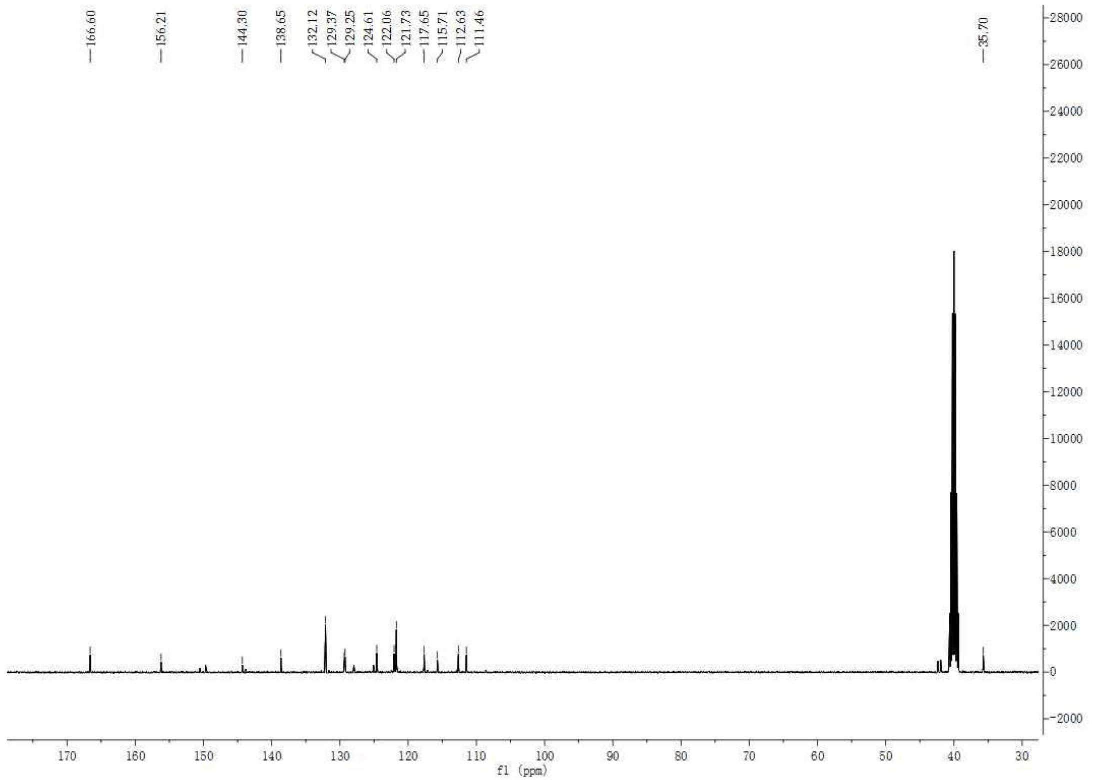

Figure S68. [13] C-NMR spectra of the target compound D14

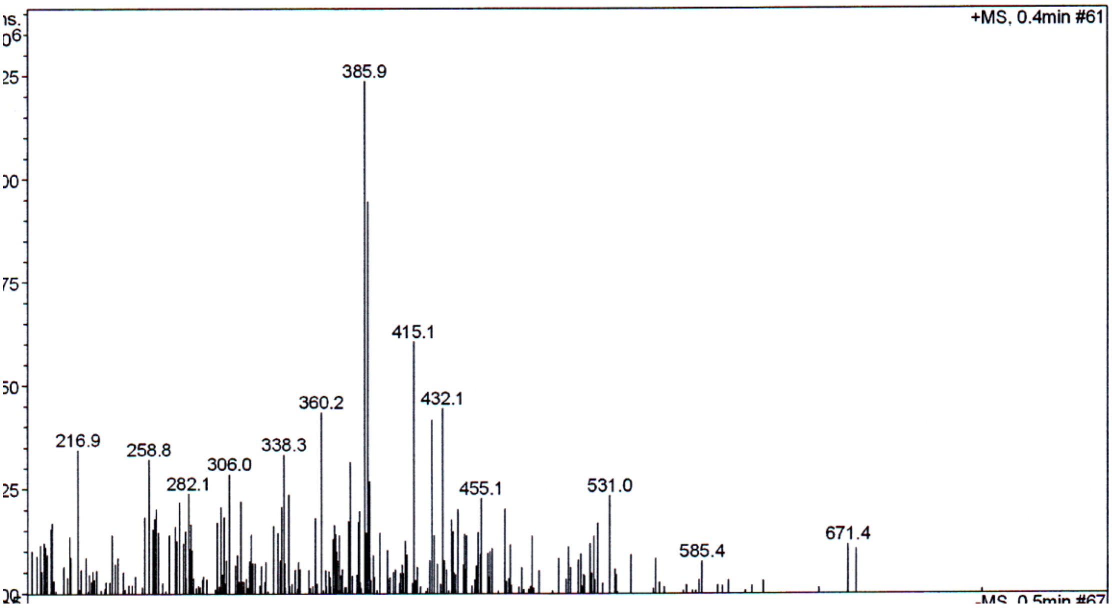

Figure S69. ESI-MS of the target compound D14

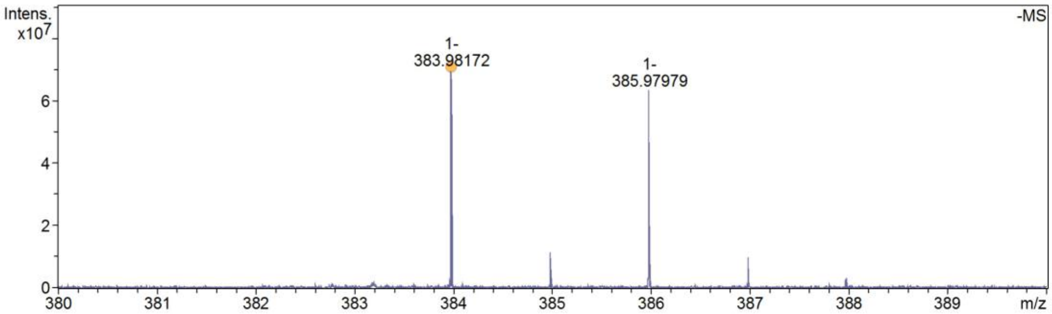

Meas. m/z	#	Ion Formula	Score	m/z	err [ppm]
383.981719	1	C17H11BrN3OS	100.00	383.981169	-1.4

Figure S70. HRMS of the target compound D14

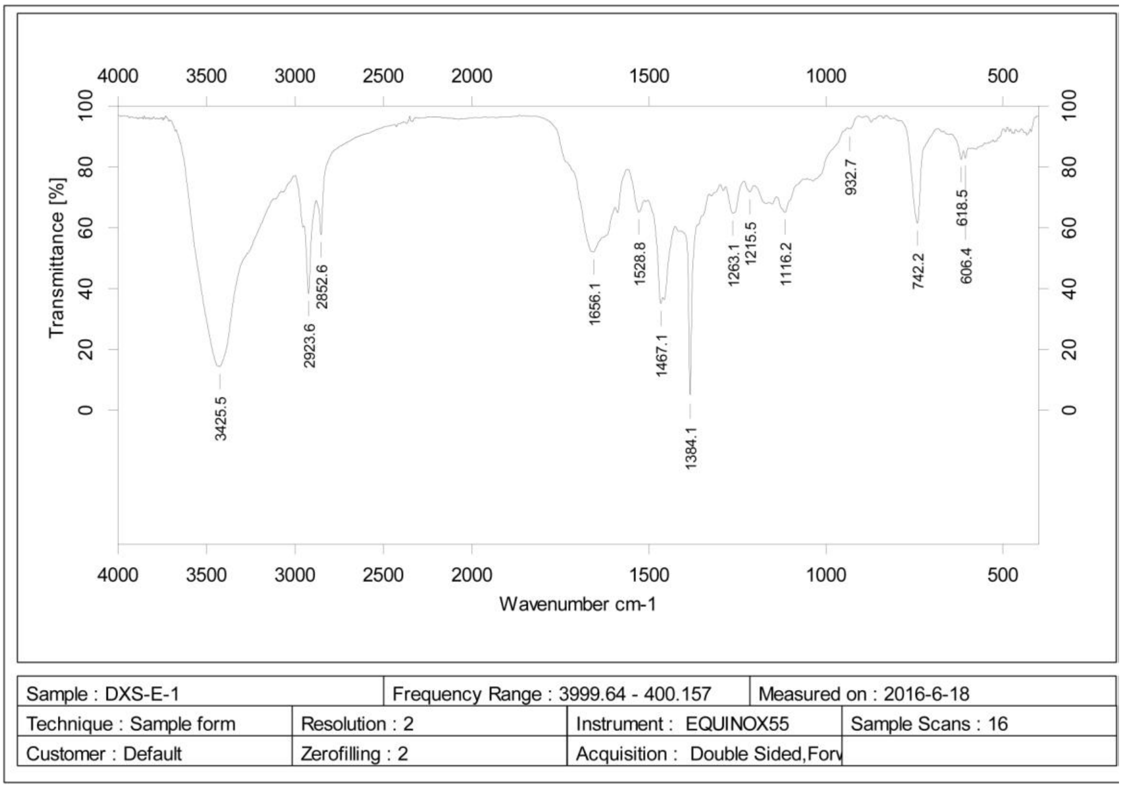

Figure S71. IR spectra of the target compound D15

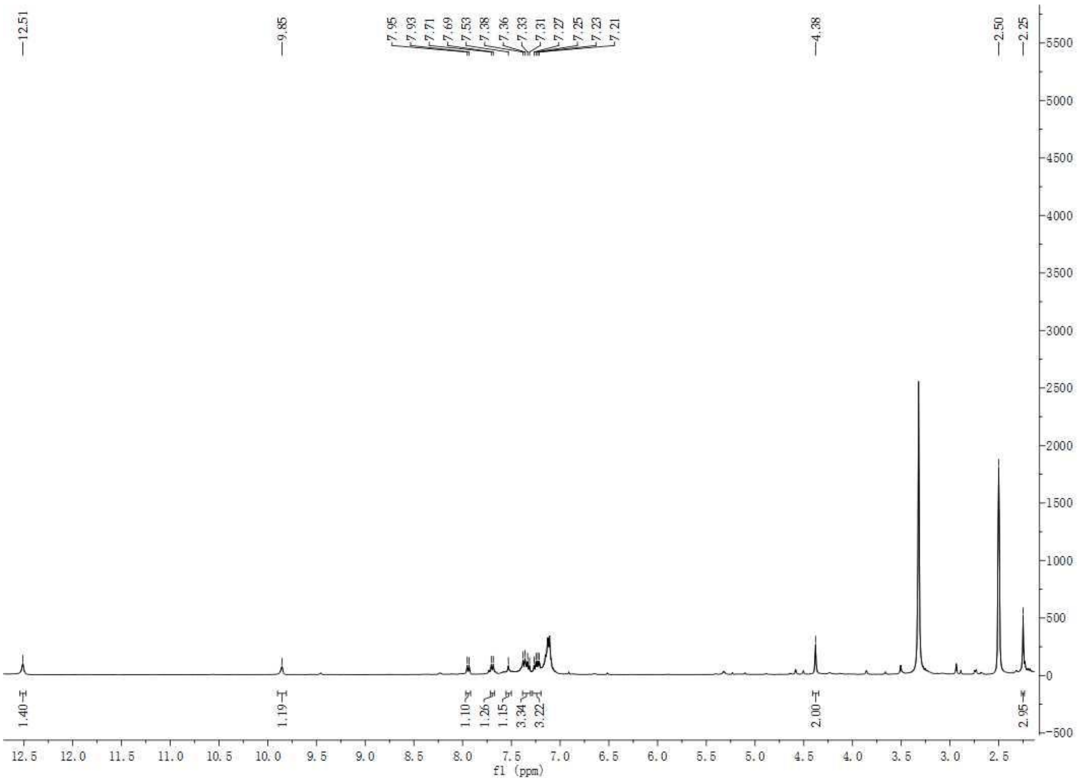

Figure S72. ^{1}H-NMR spectra of the target compound D15

molecules MDPI

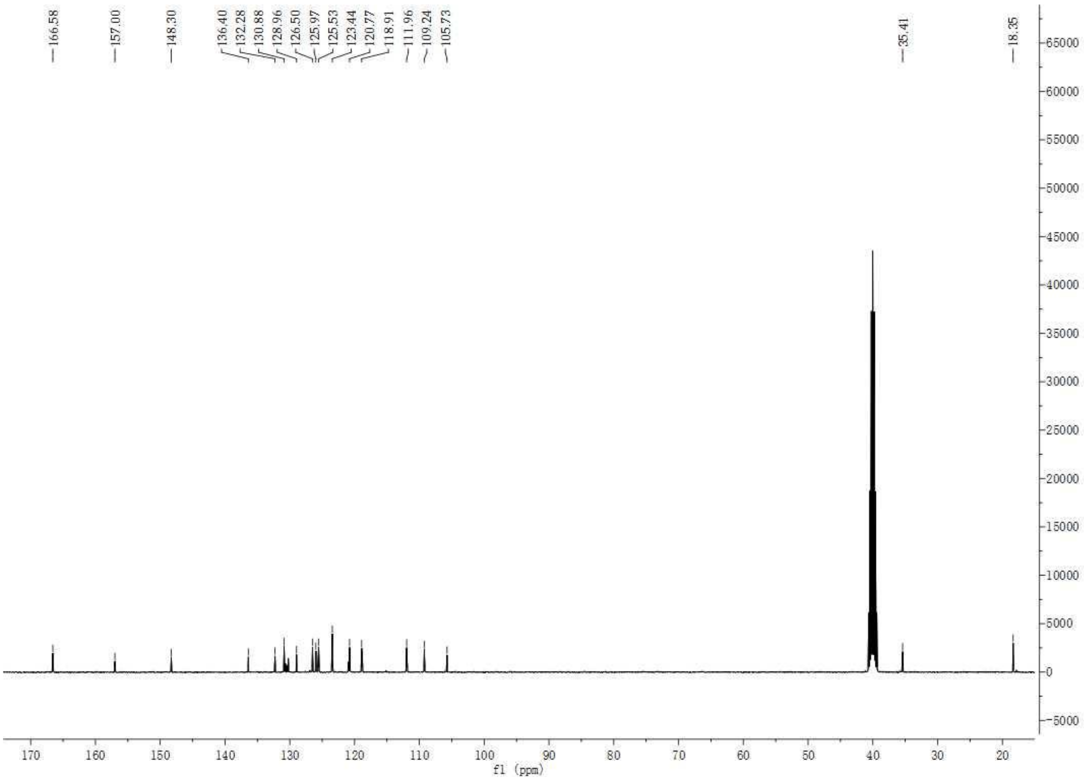

Figure S73. ^{13}C-NMR spectra of the target compound D15

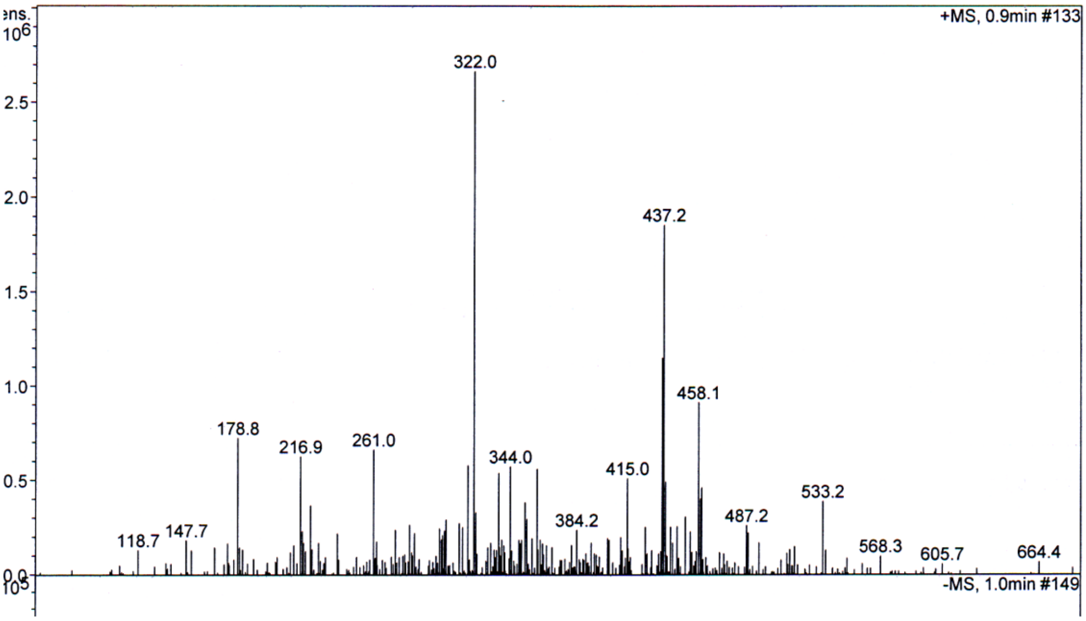

Figure S74. ESI-MS of the target compound D15

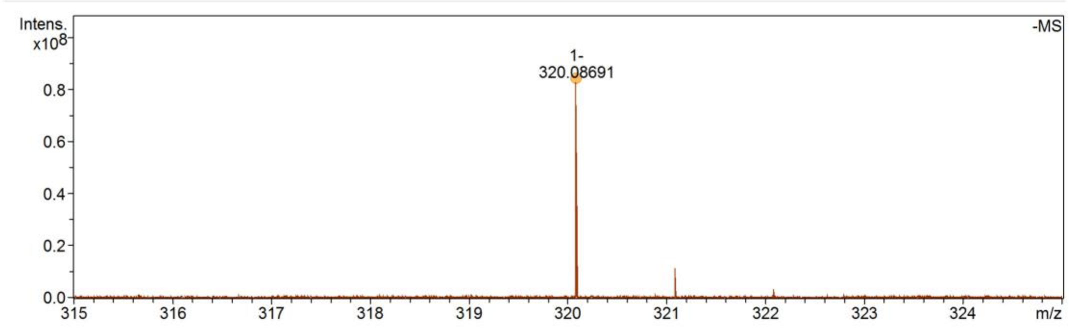

Meas. m/z	#	Ion Formula	Score	m/z	err [ppm]
320.086912	1	C18H14N3OS	100.00	320.086307	-1.9

Figure S75. HRMS of the target compound D15

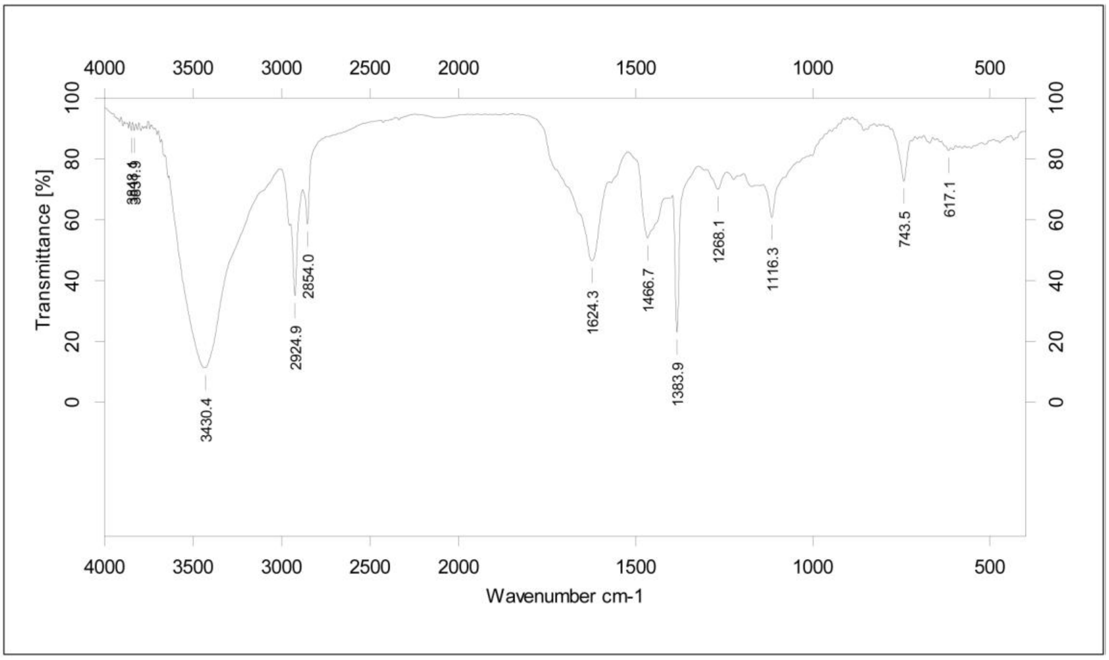

Sample : DXS-E-16	Frequency Range : 3999.64 - 400.157	Measured on : 2016-6-18	
Technique : Sample form	Resolution : 2	Instrument : EQUINOX55	Sample Scans : 16
Customer : Default	Zerofilling : 2	Acquisition : Double Sided,Forv	

Figure S76. IR spectra of the target compound D16

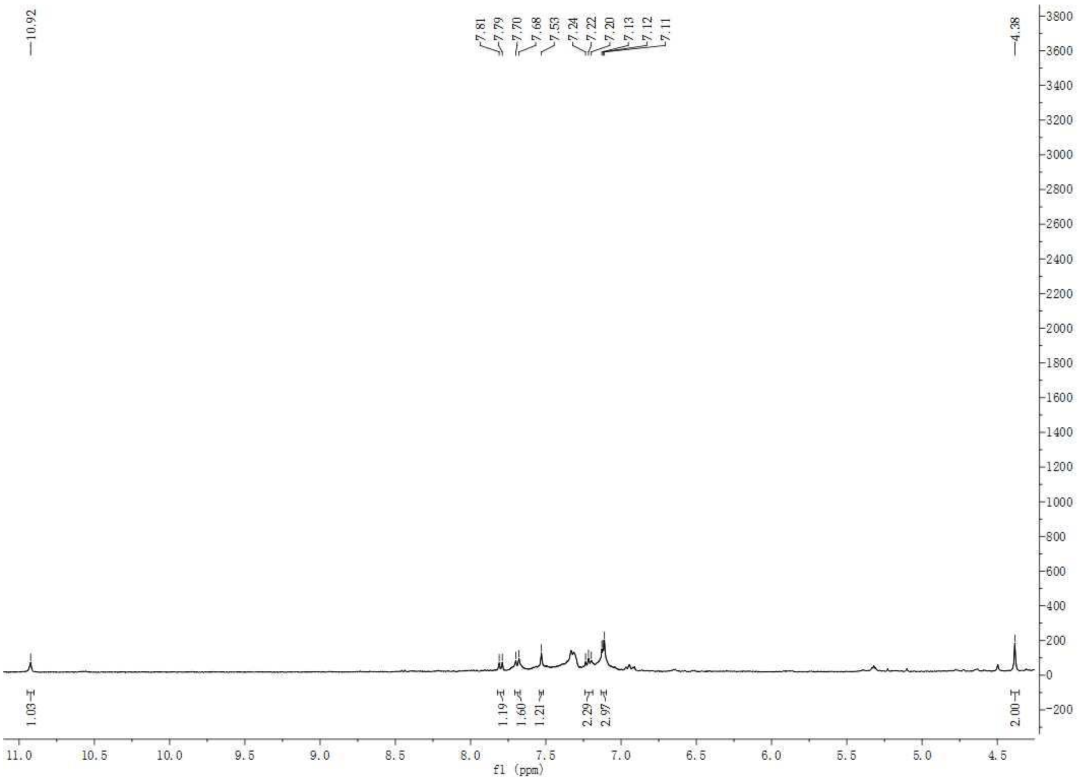

Figure S77. [1] H-NMR spectra of the target compound D16

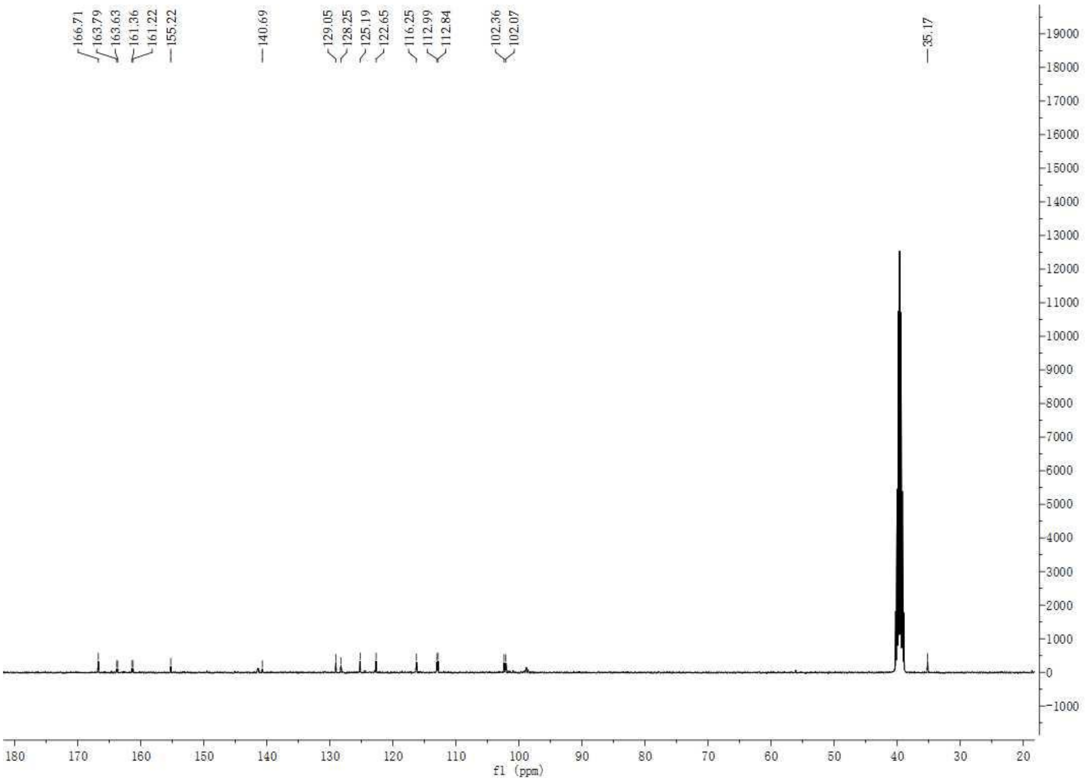

Figure S78. [13] C-NMR spectra of the target compound D16

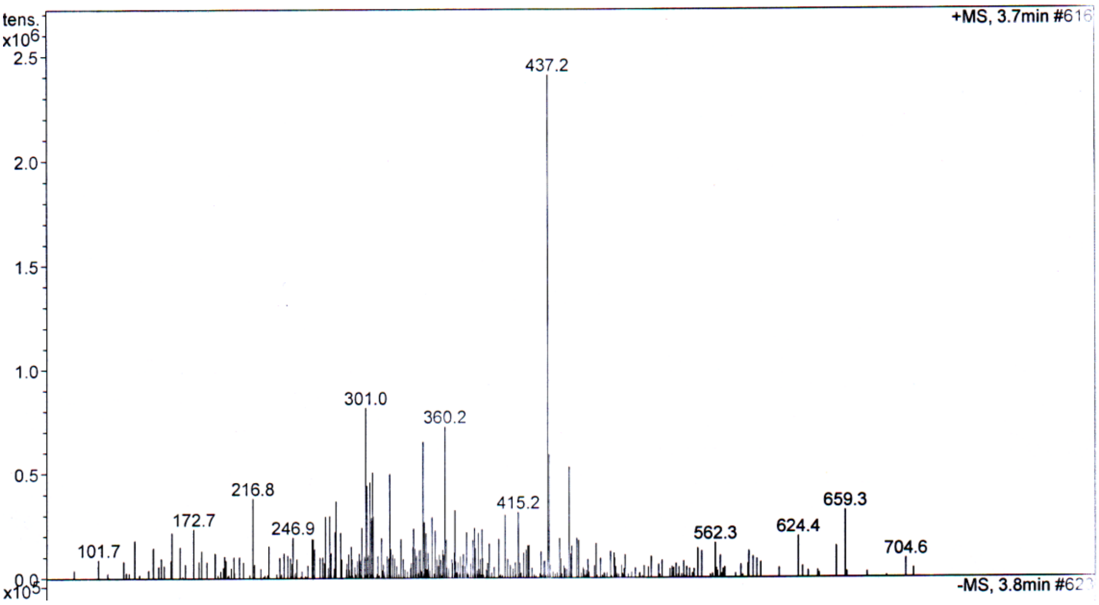

Figure S79. ESI-MS of the target compound D16

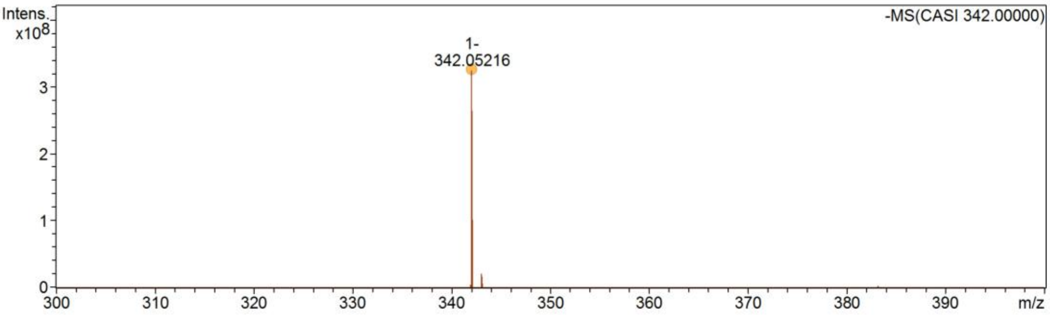

Meas. m/z	#	Ion Formula	Score	m/z	err [ppm]
342.052163	1	C17H10F2N3OS	100.00	342.051813	-1.0

Figure S80. HRMS of the target compound D16

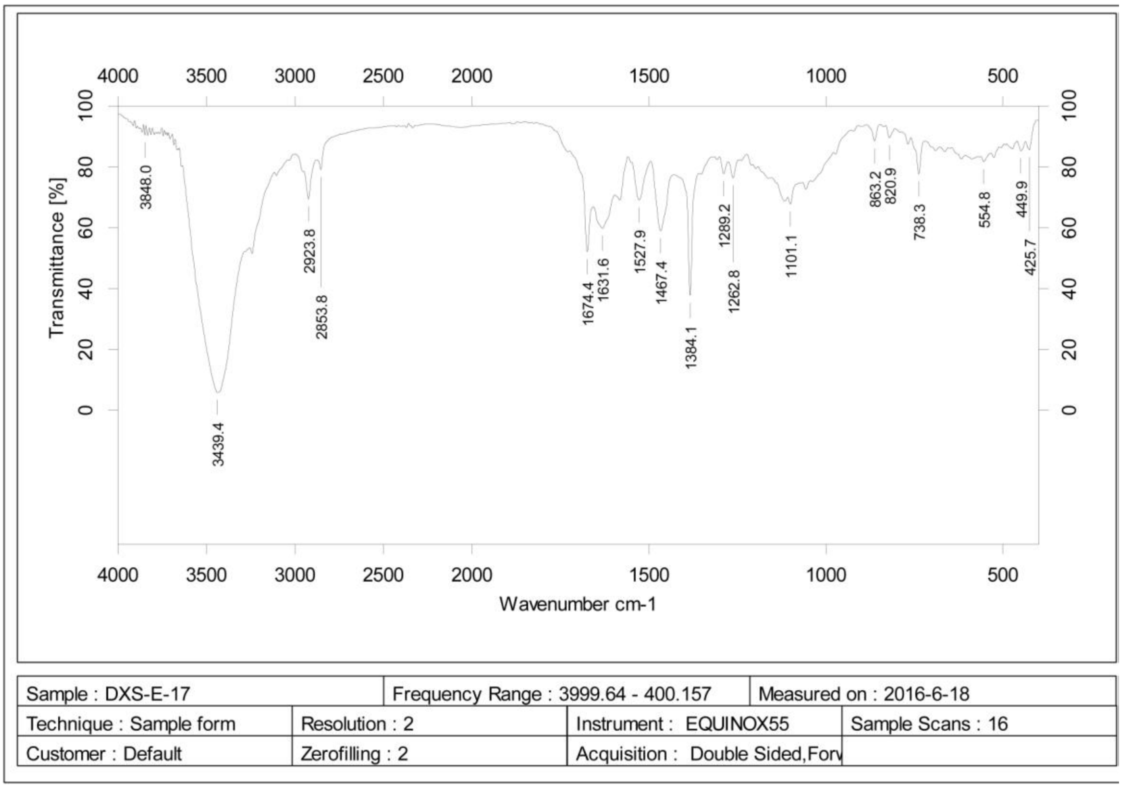

Figure S81. IR spectra of the target compound D17

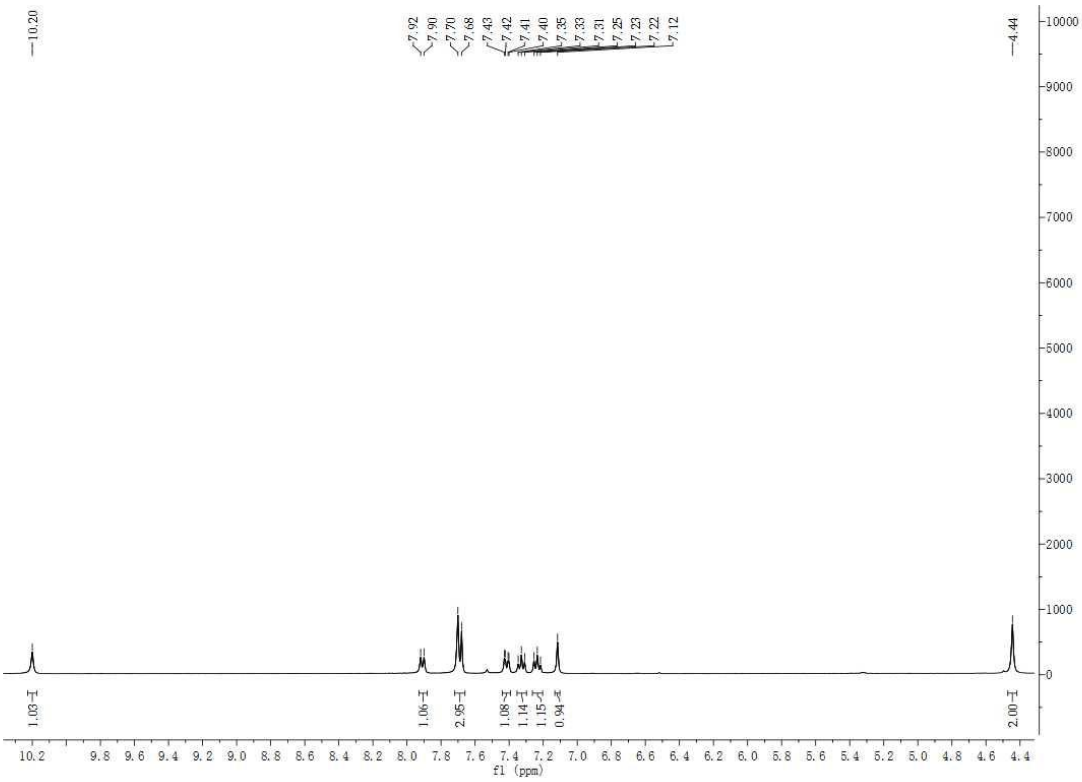

Figure S82. [1] H-NMR spectra of the target compound D17

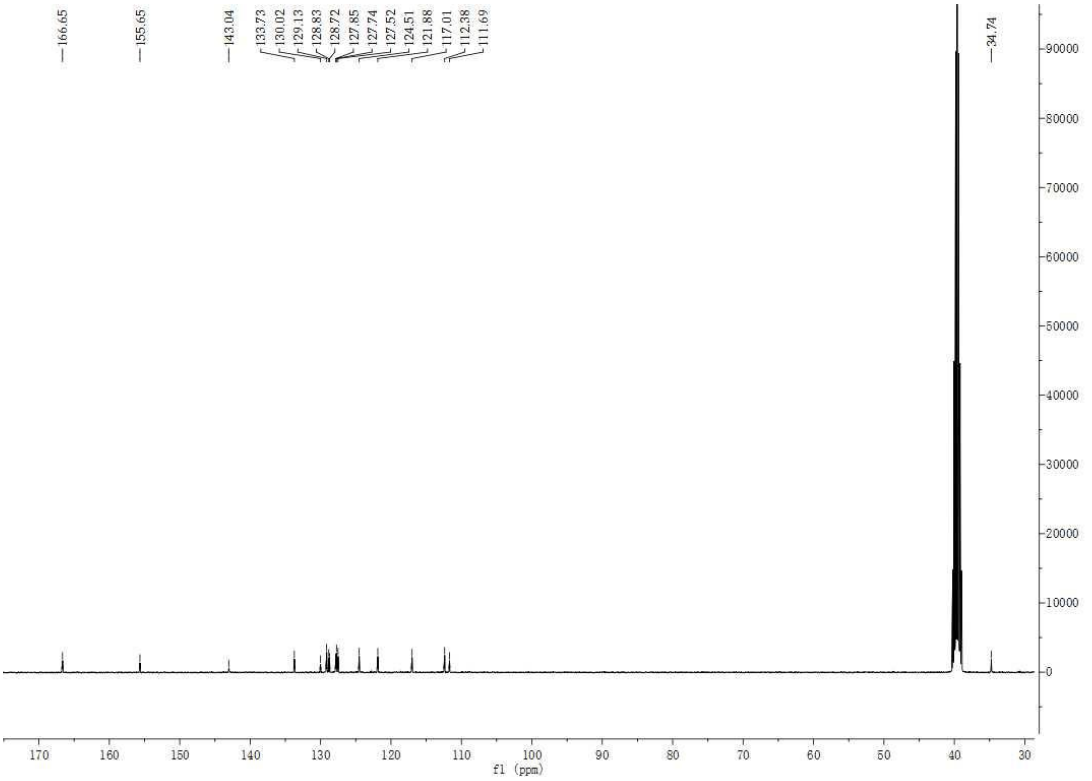

Figure S83. ^{13}C-NMR spectra of the target compound D17

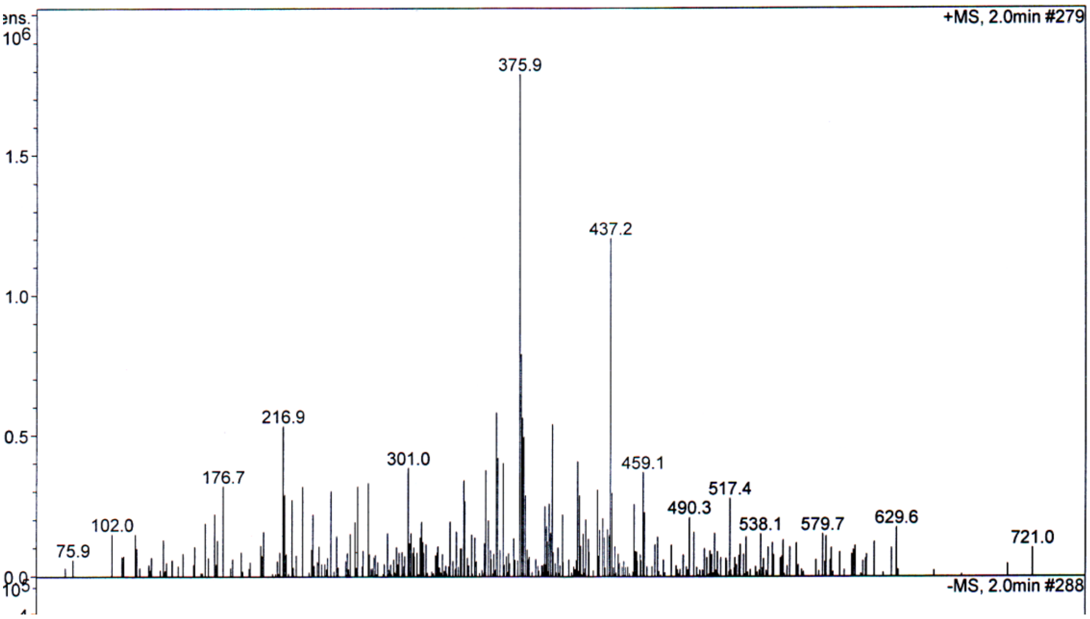

Figure S84. ESI-MS of the target compound D17

molecules

MDPI

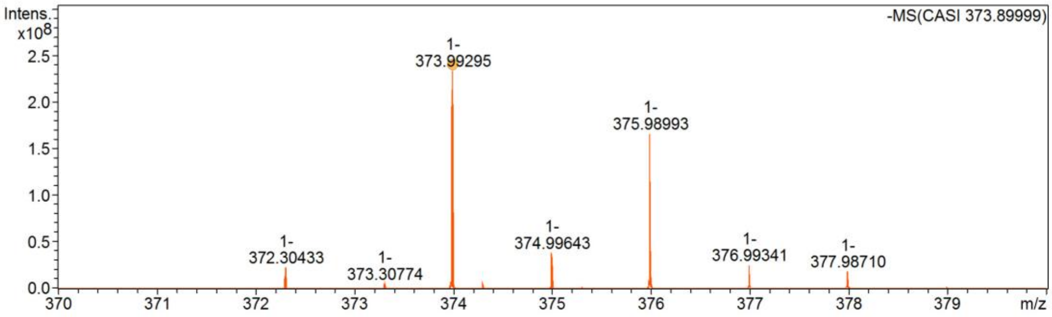

Meas. m/z	#	Ion Formula	Score	m/z	err [ppm]
373.992947	1	C17H10Cl2N3OS	100.00	373.992712	-0.6

Figure S85. HRMS of the target compound D17

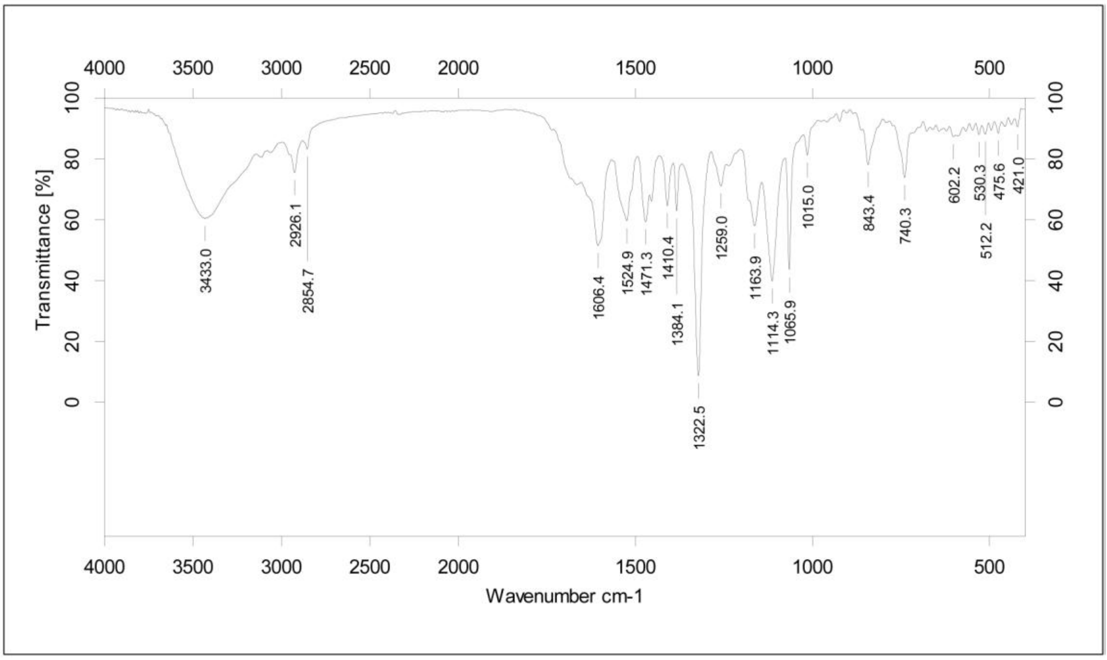

Sample : DXS-E-21	Frequency Range : 3999.64 - 400.157	Measured on : 2016-6-18	
Technique : Sample form	Resolution : 2	Instrument : EQUINOX55	Sample Scans : 16
Customer : Default	Zerofilling : 2	Acquisition : Double Sided,Forv	

Figure S86. IR spectra of the target compound D18

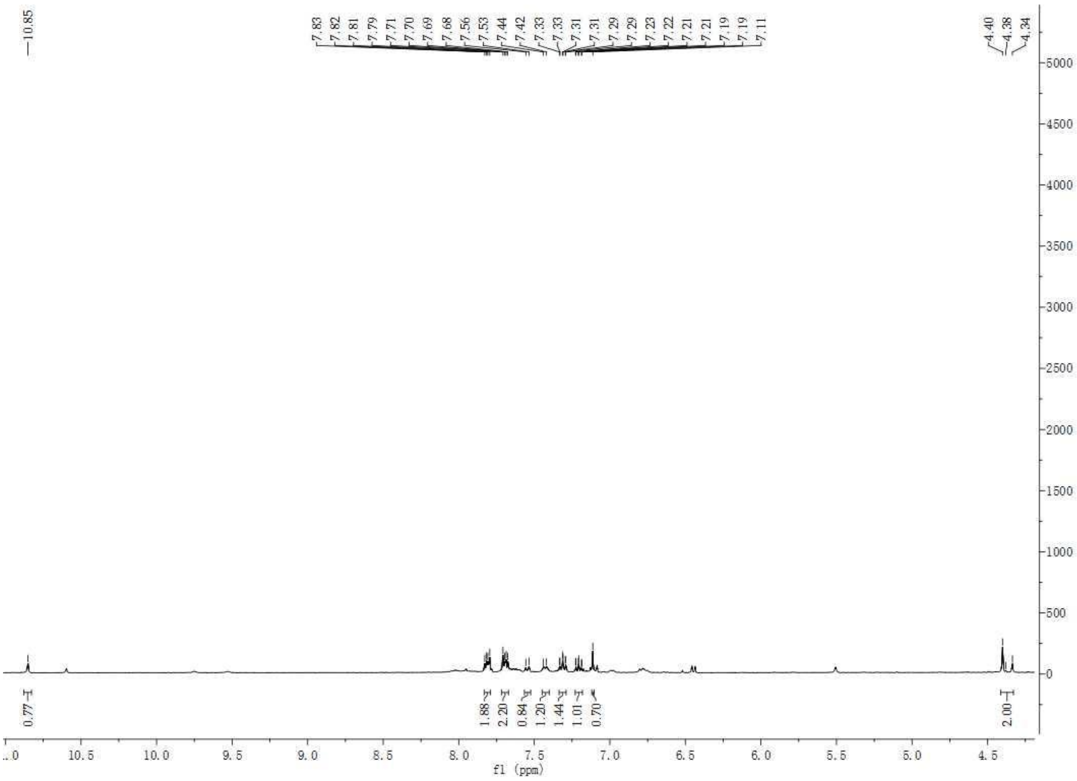

Figure S87. ^{1}H-NMR spectra of the target compound D18

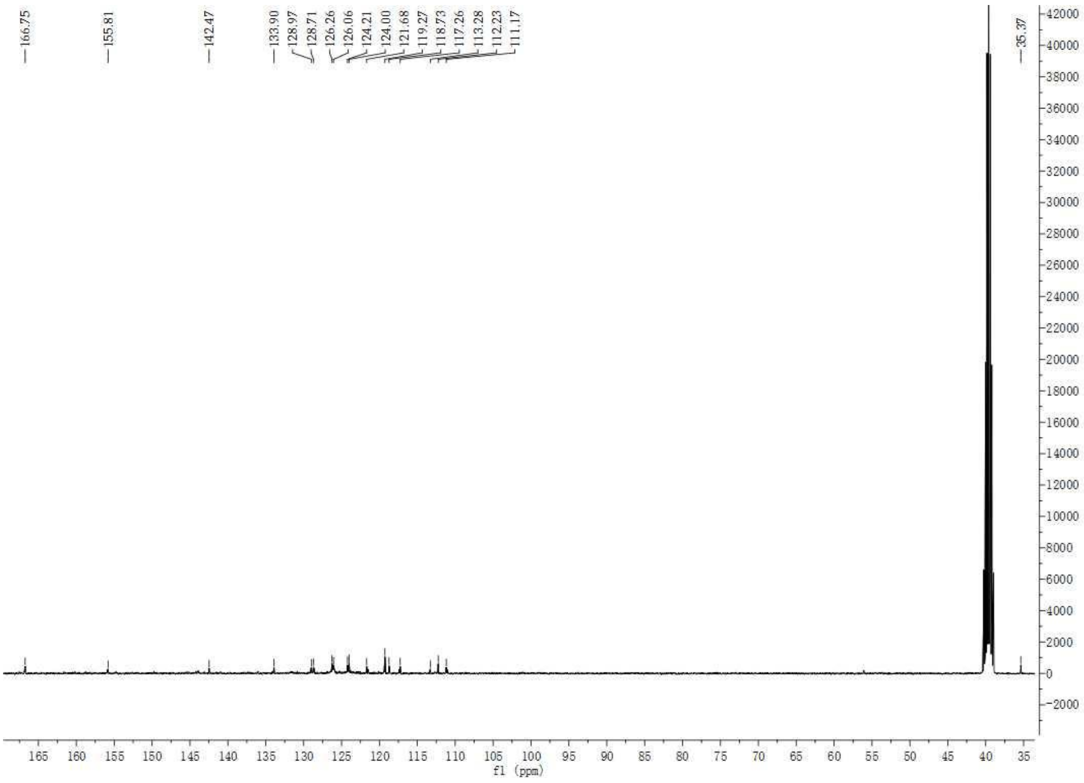

Figure S88. ^{13}C-NMR spectra of the target compound D18

molecules MDPI

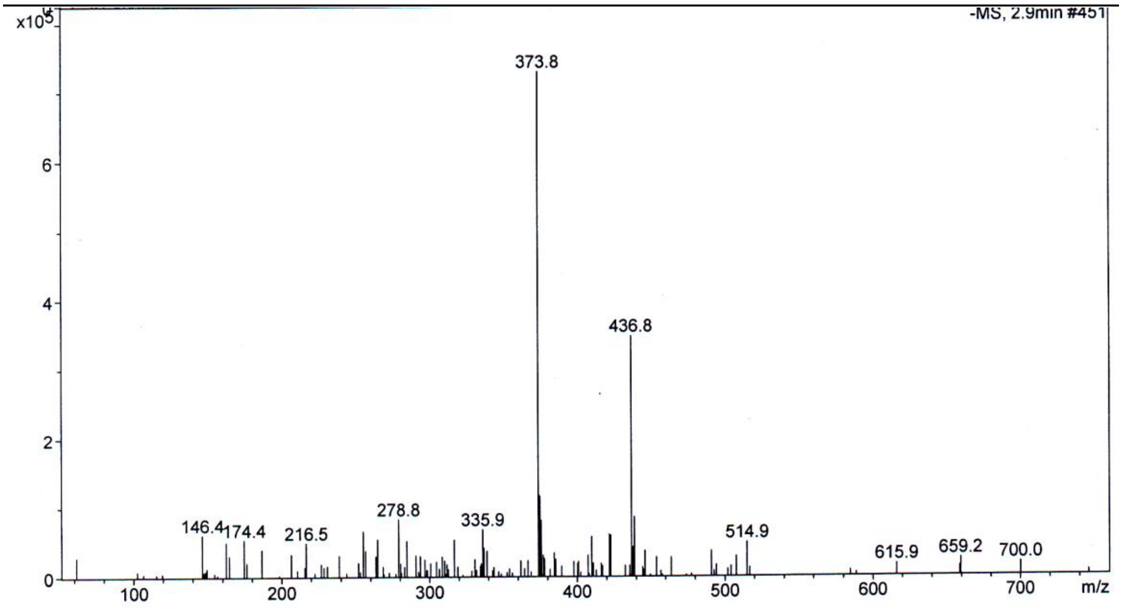

Figure S89. ESI-MS of the target compound D18

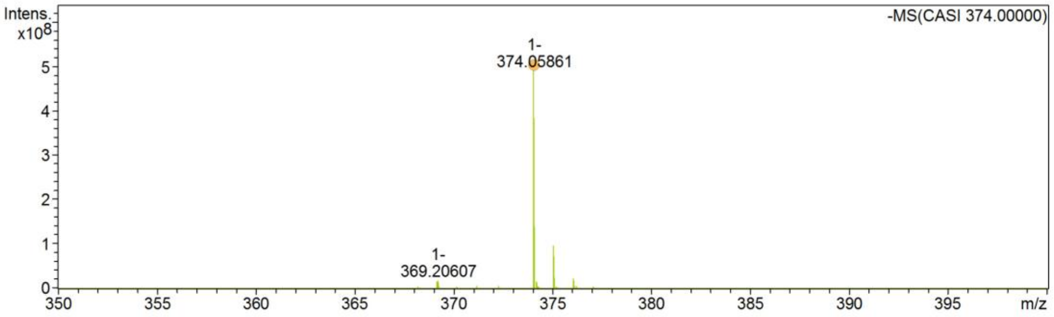

Meas. m/z	#	Ion Formula	Score	m/z	err [ppm]
374.058608	1	C18H11F3N3OS	14.23	374.058041	-1.5

Figure S90. HRMS of the target compound D18
